# Clocked stepping of an artificial protein walker along a DNA track

**DOI:** 10.1038/s41565-026-02211-3

**Published:** 2026-07-06

**Authors:** Patrik Nilsson, Neil O. Robertson, Nils Gustafsson, Roberta B. Davies, Chu Wai Liew, Aaron Lyons, Ralf Eichhorn, Cassandra S. Niman, Gerhard A. Blab, Elizabeth H. C. Bromley, Andrew E. Whitten, Anthony P. Duff, Ivan N. Unksov, Jason P. Beech, Peter Jönsson, Till Böcking, Birte Höcker, Derek N. Woolfson, Nancy R. Forde, Heiner Linke, Paul M. G. Curmi

**Affiliations:** 1 NanoLund, Lund University, Lund, Sweden; 2 Solid State Physics, Lund University, Lund, Sweden; 3 School of Physics, University of New South Wales, Sydney, New South Wales Australia; 4 School of Biotechnology and Biomolecular Sciences, University of New South Wales, Sydney, New South Wales Australia; 5 Department of Physics, Simon Fraser University, Burnaby, British Columbia Canada; 6 Nordita, Royal Institute of Technology and Stockholm University, Stockholm, Sweden; 7 Department of Physics, University of Durham, Durham, UK; 8 Australian Nuclear Science and Technology Organisation, Lucas Heights, New South Wales Australia; 9 Physical Chemistry, Lund University, Lund, Sweden; 10 Department of Molecular Medicine, School of Biomedical Sciences, University of New South Wales, Sydney, New South Wales Australia; 11 EMBL Australia Node in Single Molecule Science, School of Biomedical Sciences, University of New South Wales, Sydney, New South Wales Australia; 12 Department of Biochemistry, University of Bayreuth, Bayreuth, Germany; 13 School of Chemistry, University of Bristol, Bristol, UK; 14 School of Biochemistry, University of Bristol, Bristol, UK; 15 Max Planck-Bristol Centre for Minimal Biology, University of Bristol, Bristol, UK; 16 NNF Center for Protein Design, University of Copenhagen, Copenhagen, Denmark; 17 Science for Life Laboratory, Physics Department, Lund University, Lund, Sweden; 18 Present Address: Debye Institute for Nanomaterials Science and Department of Physics, Utrecht University, Utrecht, The Netherlands

**Keywords:** Nanobiotechnology, Nanobiotechnology

## Abstract

Molecular motors are fundamental to life because they transduce free energy into mechanical work, a capability rooted in the chemical and structural complexity of their constituent proteins. Although motors based on small molecules and DNA have been developed, the creation of an artificial protein motor has remained an elusive goal in synthetic biology. Here we report the realization of an artificial, externally controlled protein motor, termed Tumbleweed (TW). TW was engineered using a modular design strategy that combines proteins with well-characterized properties to produce emergent motor function and directionality. TW comprises three ‘legs’, each containing a ligand-gated DNA-binding domain that enables selective interaction with specific sites along a DNA track. Using single-molecule fluorescence assays in conjunction with a programmable microfluidic device, we show that TW takes directional 16 nm steps along a designed DNA substrate in response to a defined sequence of ligand inputs. Moreover, both the timing and direction of stepping can be precisely controlled on a timescale of seconds. This approach provides a versatile platform for engineering dynamic and sophisticated protein-based nanomachines, as well as for probing the physical principles governing protein walkers with precisely defined architectures.

## Abstract (web summary)

A synthetic three-footed protein walker assembled from non-motor protein domains takes directional steps along a DNA track powered by Brownian ratchet motion driven by free energy transduction from microfluidically delivered ligands.

## Main

The ability to transduce chemical energy to mechanical work^1,2^ is essential for life^3–8^. Nature has evolved molecular motors that achieve this task with remarkable efficiency and precision, using engineering principles that are starkly different to our human-made engines. This high level of performance has been enabled by the chemical and structural complexity of proteins.

What if we could build artificial protein motors from the bottom up? Doing so would give us precise control over design details and help unravel how motor function emerges from the interplay of distinct components. Designing and realizing an artificial protein that functions as a walker or motor is therefore a major goal in synthetic biology and nanotechnology^9^.

Despite recent progress, this goal has remained elusive. Major efforts in synthetic systems have yielded motors made from small molecules^10^ and DNA^11,12^. However, none of these designed molecular machines have achieved the processivity, speed and efficiency found in natural protein motors^13^. De novo protein design has recently created components that could be incorporated into motors such as switchable assemblies^14,15^, hinge proteins^16^, as well as an axle-rotor protein^17^ and a non-directional protein walker^18^. However, the outstanding challenge remains to couple and coordinate flexible protein components so that surface-binding and directed motion are realized in concert with energy transduction to achieve motor function.

**Fig. 1:**
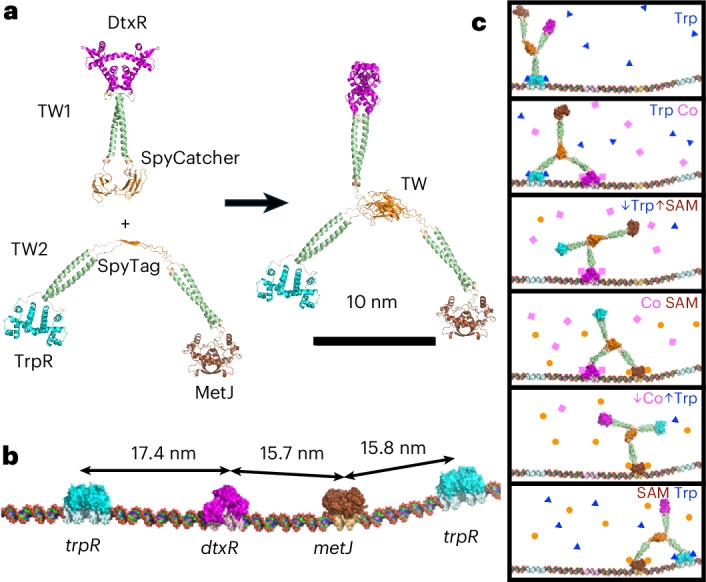
The TW artificial clocked-walker protein concept. **a**, We constructed TW from two homodimeric proteins, TW1 and TW2. TW1 comprises the DtxR repressor, a geminin helix and the SpyCatcher protein. TW2 comprises the TrpR repressor, a geminin helix, the SpyTag, another geminin helix and the MetJ repressor. Combining TW1 and TW2, the SpyTag/SpyCatcher system covalently assembles TW. **b**, To achieve unidirectional motion, we used a DNA track with an ordered series of cognate binding sites for each of the three TW feet (shown docked): *trpR*–*dtxR*–*metJ*, which can repeat indefinitely, and are arranged so that TW remains on the same face of the track. **c**, Directional motion was controlled by time-dependent bathing of TW in ligand solutions. In the presence of tryptophan (top panel; Trp, blue triangle), TW is bound by TrpR foot (cyan). Upon adding Co^2+^ (pink squares, second panel from top), the DtxR foot (magenta) also binds to the track adjacent to the TrpR foot. When the concentration of Trp decreases (third panel from top), the TrpR foot releases the track. When adding *S*-adenosyl methionine (fourth panel from top; SAM; orange circle), the MetJ foot (brown) binds adjacent to the DtxR foot. When the Co^2+^ concentration is decreased, the DtxR foot releases (second panel from bottom). Finally, upon re-addition of Trp, the TrpR foot binds adjacent to the MetJ foot (bottom panel). Note that TW and the track were designed so that only one site is reachable for each binding foot (for example, the near but not the far *trpR* site in the bottom panel).

Here we present a functional, chemically powered, artificial clocked-walker protein that travels directionally along a DNA track under the control of microfluidically delivered ligands. It is created through a modular, bottom-up approach that combines biological and designed protein elements that individually lack inherent motor function. Our design strategy overcomes challenges in de novo protein design by assembling well-characterized functional modules: connecting ligand-controlled track-binding feet and legs with appropriate flexibility. The resulting clocked-walker protein, which we named Tumbleweed (TW)^19^, can take multiple, 16 nm steps directionally along a DNA track under external control on a timescale of seconds (Fig. 1).

## Modular concept for a clocked-walker protein

TW is enabled by the following conceptual ingredients^19^ (Fig. 1). First, TW has three distinct feet, each made from different repressor proteins that bind to cognate DNA sites in the presence of their respective ligand: TrpR and L-tryptophan (Trp); DtxR and Co^2+^; and MetJ and *S*-adenosyl-L-methionine (SAM). These feet are connected to a central hub by α-helical coiled-coil legs, resulting in a three-legged walker (Fig. 1a).

Second, to ensure unidirectional motion, the DNA track contains specific binding sites for TW feet in a unique order: *trpR*, *dtxR*, *metJ* (italics with lowercase first letter represent cognate sites on DNA). These binding sites repeat for the length of the track (Fig. 1b), enabling directionality (Fig. 1c). We designed DNA tracks to space the binding sites such that each TW foot would bind on the same side of the DNA (Fig. 1b). We adjusted the spacing between sites relative to the stride of TW so that two of its feet could bind simultaneously to adjacent sites but not to non-adjacent sites (assuming a straight DNA track).

Third, to control TW motion, we bathed TW in solutions comprising pairs of controlling ligands in a cyclical, time-dependent manner: with (Trp + Co^2+^), then (Co^2+^ + SAM), then (SAM + Trp). By doing so, TW cycles through states in which it is first bound to the track via TrpR and DtxR, then via DtxR and MetJ, and finally via MetJ and TrpR (Fig. 1c). In between these relatively stable, two-foot-bound states, TW will transit through more short-lived, single-foot-bound states (Fig. 1c). The direction of motion can be controlled by altering the order of ligand pairs.

The exchange of ligands supplies TW with the free energy to power its unidirectional motion. Each DNA-binding module binds its respective ligand when the ligand concentration is high (high chemical potential) and eventually dissociates and releases its controlling ligand when the ligand concentration is low (low chemical potential). This is the same mechanism used to power rotary motors such as the F_0_-ATPase^20^ and MotAB^21^. As each of TW’s steps is facilitated by diffusive motion, TW is a chemically powered Brownian ratchet^22^.

## Realizing the TW concept

**Fig. 2:**
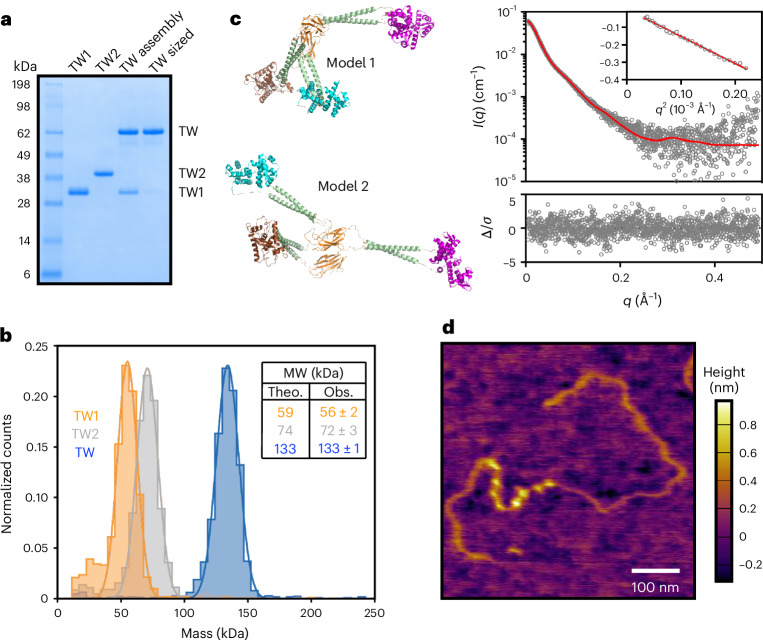
Assembly and characterization of TW. **a**, SDS–PAGE showing the covalent TW assembly and purified TW after SEC (TW sized). TW sized protein was used for all remaining experiments. **b**, Mass photometry characterization of purified TW1, TW2 and TW. Insets: the theoretical (Theo.) and observed (Obs.) molecular weights (mean ± s.e.m., *n* = 4) for each protein species. **c**, SEC-SAXS analysis of TW. Top right shows the SAXS intensity as a function of *q* (momentum transfer in Å^−1^). Inset: a Guinier plot of the data; data were fit to an ensemble of models using the programme Multi-FoXS. The best fit (top right, red line) as determined by *χ*^2^ was obtained using two conformers, one L-shaped (Model 1, top left) and one extended (Model 2, bottom left), weighted at 0.725 and 0.275, respectively. The error-weighted residual plot (lower right panel) shows no significant systematic deviations between the model and the experimental data. **d**, AFM image showing eight TWs bound in the presence of Trp to eight repeats of a *metJ*–*trpR*–*dtxR* track inserted between flanking sequences. Source data

As heterotrimeric proteins are uncommon, we designed and assembled TW from two separate components (TW1 and TW2) using the SpyTag/SpyCatcher system^23^ (Fig. 1a). TW1 comprised DtxR, the α-helical domain from the geminin coiled-coil, and SpyCatcher, each connected by flexible Gly-Ser linkers. TW2 comprised TrpR, the geminin helix, SpyTag, a second copy of the geminin helix and MetJ, each connected by Gly-Ser linkers. We produced each component by expressing synthetic genes in *Escherichia coli* and purifying the His-tagged proteins using immobilized metal affinity chromatography (Fig. 2a).

TW1 and TW2 formed homodimeric proteins that were purified to homogeneity (Fig. 2a). We used mass photometry to determine the size of each molecule, which was consistent with the expected mass (Fig. 2b and Supplementary Table 1). Mature TW was then generated by mixing TW1 and TW2, with a slight excess of TW1 (1.2:1). We confirmed the covalent TW product by sodium dodecyl sulfate polyacrylamide gel electrophoresis (SDS–PAGE; Fig. 2a). The product was purified by size-exclusion chromatography (SEC). Mass photometry confirmed that the size was consistent with the expected mass (Fig. 2b and Supplementary Table 1).

To verify the structural integrity of TW, we collected SEC-coupled small-angle X-ray scattering (SEC-SAXS) data (Fig. 2c). The observed radius of gyration (*R*_*g*_ Guinier 67.7 ± 0.6 Å; *R*_*g*_ *P*(*r*) 69.1 ± 0.4 Å) was consistent with an extended protein (Supplementary Fig. 1). Given the hinge flexibility of TW, we anticipated an ensemble of structures in solution. We fit the experimental data using Multi-FoXS^24^, allowing the programme to bend AlphaFold2-generated models^25^ of TW via six hinge points linking legs to both the hub and DNA-binding modules. We obtained a best fit with two conformers: one L-shaped (72.5%; Model 1, Fig. 2c, top left) and one extended (27.5%; Model 2, Fig. 2c, bottom left). Adding further conformations only marginally improved the fit and the additional conformers clustered with the original two.

To visualize TW binding to a DNA track, we produced a track with eight copies of *metJ*–*trpR*–*dtxR* inserted into the pYIC plasmid^26^. We digested the plasmid to generate a linear DNA track, mixed it with TW in the presence of 10 mM Trp and visualized it using atomic force microscopy (AFM). Images revealed eight bright, evenly spaced features along the DNA (Fig. 2d). The contour lengths of the DNA flanking the eight bright features (representing bound TWs) are consistent with the location of the inserted (*metJ*–*trpR*–*dtxR*)_8_ track.

**Fig. 3:**
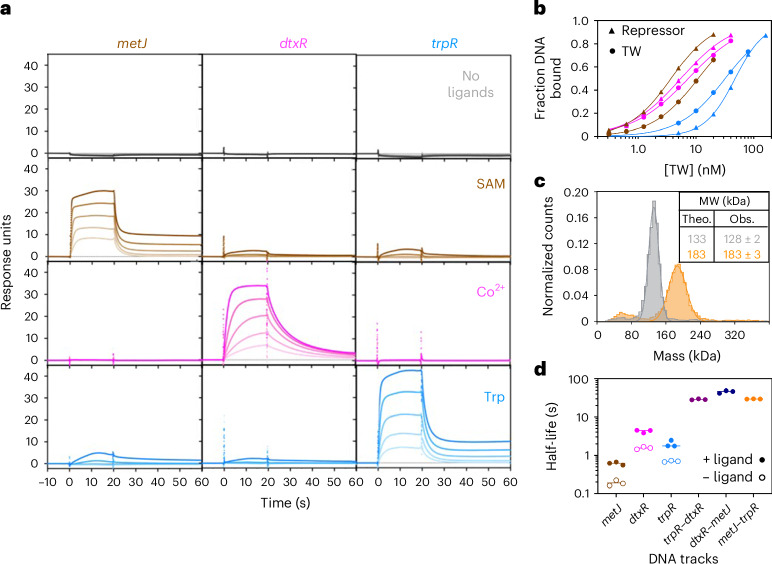
Optimizing TW:DNA track interaction. **a**, Panel of SPR sensorgrams titrating 5 nM, 10 nM, 20 nM, 40 nM and 80 nM TW onto DNA oligonucleotides containing single cognate sites (*metJ*, *dtxR* and *trpR* sites) in the presence or absence of controlling ligands showing the intended TW binding of each foot (in the presence of ligands) to the respective cognate DNA, with minimal off-site non-specific binding. **b**, Representative binding curves fitting the Hill equation to average steady-state SPR responses. Curves show binding to single-site tracks containing individual cognate sites (brown, *metJ*; magenta, *dtxR*; cyan, *trpR*) for both TW (circles) and individual repressor dimers (triangles) in the presence of the controlling ligand. **c**, Mass photometry data comparing the distribution of estimated molecular masses for the complex of TW and a duplex DNA containing the *metJ* and *trpR* sites (TW:*metJ–trpR* complex) in the absence of ligands (grey) and in the presence of SAM and Trp (orange). The observed (Obs.) molecular masses of 128 ± 2 kDa (mean ± s.e.m.) in the absence of ligands and 183 ± 3 kDa in the presence of SAM and Trp are in excellent agreement with the theoretical (Theo.) masses of 133 kDa and 183 kDa, respectively. **d**, Apparent half-lives of different TW:DNA complexes as calculated from fitted *k*_off_ values determined by SPR experiments in which TW was bound to DNA in the presence (filled circles) or after removal (open circles) of ligands. Dissociation was triggered by adding an excess of free competing specific DNA in the presence or absence of ligands. Controlling ligands correspond to the respective DNA tracks, as indicated. Circles represent the individual values from replicate experiments. Source data

**Extended Data Fig. 1:**
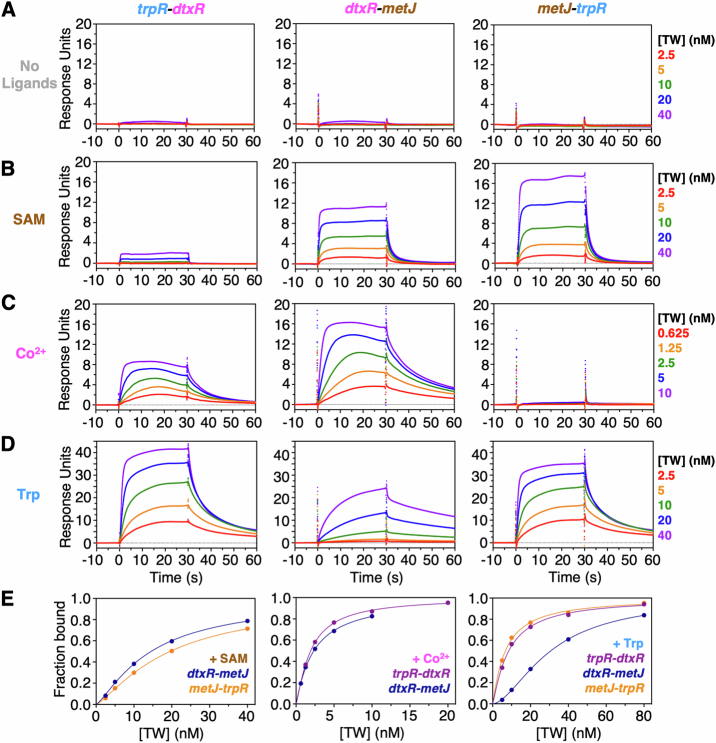
Tumbleweed binding double-site DNA in the presence of single ligands, by SPR. TW was titrated onto DNA containing two repressor cognate sites (**A**) in the absence of ligand, and in the presence of (**B**) SAM, (**C**) Co^2+^ or (**D**) Trp. The DNA sites used in these experiments are named above the figures, with sequences reported in Supplementary Table 5. (**E**) Representative data for steady state SPR binding isotherms of TW binding to double-site DNA fitted to the Hill equation. The ligands and DNA used in these experiments are as per the labels within the figure panels. The fitted parameters for TW binding to the double site DNA are reported in Extended Data Table 1. Source data

**Extended Data Fig. 2:**
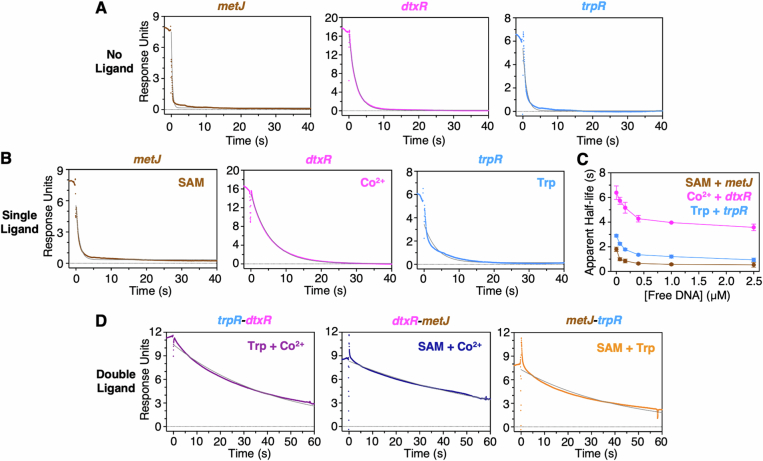
Dissociation kinetics of tumbleweed, by SPR. Representative dissociation experiments in which TW was loaded with the relevant ligand onto DNA containing either one or two repressor cognate sites. Ligands used in each experiment are labelled within the figure. Curves were fit to a single exponential decay function, with the apparent off-rates reported in Extended Data Table 2. (**A**) Dissociation in the presence of DNA but absence of ligand. (**B**) Dissociation in the presence of both DNA and single ligands. (**C**) Apparent half-lives of TW bound to single site DNA in the presence of single ligands with titration of free DNA. Points represent the mean ± standard deviation from replicate experiments (n = 3–4). Lines connect the points from different experiments. (**D**). Dissociation in the presence of double ligands and two free oligonucleotides, each containing a single relevant repressor cognate site. Across all experiments, dissociation was triggered by injection of a solution containing 1 µM free DNA containing the same cognate site as the DNA bound to the SPR chip surface. Free DNA sequences lacked an SPR anchor overhang and therefore could not bind to the SPR surface. The DNA sites used in these experiments are named above the figures, with sequences reported in Supplementary Table 5. Source data

For TW to function, we needed to ensure that each of its feet only binds to its cognate DNA sites with high affinity (*K*_d_ = 1–100 nM) and only with its respective controlling ligand. To this end, we characterized TW binding to cognate DNAs in different ligand conditions using surface plasmon resonance (SPR; Fig. 3a, Extended Data Figs. 1 and 2, and Supplementary Fig. 2).

SPR sensorgrams for TW binding to DNA containing single cognate binding sites showed that, in the absence of ligands, TW bound minimally to DNA (Fig. 3a, top row). However, when we added each ligand, TW bound to the appropriate cognate site as intended, with minimal off-site binding (Fig. 3a, bottom three rows). The dissociation constants (*K*_d_) for TW binding to single-site DNA ranged from 10 nM to 30 nM (Fig. 3b and Extended Data Table 1), similar to that of individual repressor proteins (Fig. 3b, Extended Data Table 1 and Supplementary Fig. 2).

To further characterize specificity, we titrated TW onto two-site DNA tracks (Extended Data Fig. 1). In the absence of ligands, TW did not bind to any two-site track, where [TW] was varied from 2.5 nM to 40 nM (Extended Data Fig. 1a). In this concentration range, no non-specific binding was observed to a track containing *metJ* and *trpR* when Co^2+^ was present (Extended Data Fig. 1c). A low level of non-specific binding was observed in the presence of SAM on a track containing *trpR* and *dtxR* sites when [TW] >20 nM (Extended Data Fig. 1b). The highest degree of non-specific binding by TW was observed in the presence of the Trp ligand (Extended Data Fig. 1d). Titration of TW onto a track containing *metJ* and *dtxR* sites, in the presence of appropriate ligands, either SAM or Co^2+^, showed clear, specific binding of TW (Extended Data Fig. 1b,c,e), while increasing levels of non-specific binding were observed in the presence of non-cognate ligand Trp (Extended Data Fig. 1d,e). However, the apparent affinity of off-target binding to the *dtxR*–*metJ* track in the presence of Trp was more than threefold lower than the on-target affinities (Extended Data Table 1), and for concentrations of TW below 10 nM, non-specific binding to this track was minimal compared with specific binding (Extended Data Fig. 1d,e).

**Extended Data Fig. 3:**
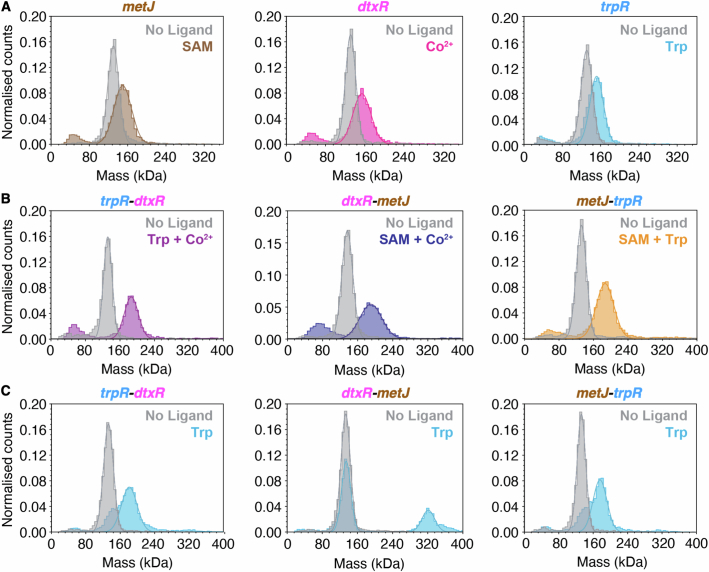
Tumbleweed forms monodisperse 1:1 complexes with both single- and double-site DNA, by mass photometry. TW:DNA mixtures were subjected to mass photometry to determine the size of complexes where resulting main mass distributions were fit to a Gaussian function, with molecular masses for each complex reported in Supplementary Table 1. (**A**) TW binding to single-site DNA in the absence and presence of single ligands. The observed molecular masses of the TW:single-site complexes (150–154 kDa) were in good agreement with the theoretical molecular masses (155–161 kDa). (**B**) TW binding to double-site DNA in the absence and presence of double ligands. The observed molecular masses of the TW:double-site complexes (183–187 kDa) were in excellent agreement with the theoretical molecular masses (182–188 kDa). (**C**) TW binding double-site DNA in the absence and presence of Trp. The observed molecular masses of the main peak of TW:double-site tracks containing a *trpR* site complexes (178–182 kDa, left and right panels) were in excellent agreement with the theoretical molecular masses (183–188 kDa). TW formed a 316 ± 3 kDa (average ± SEM, middle panel) complex with the *dtxR-metJ* track in the presence of Trp, in excellent agreement with a 2:1 TW:DNA complex (theoretical mass 315 kDa). Small mass peaks (<80 kDa) can be seen in the mass distribution histograms. Signal interference by the ligand, SAM, resulted in a small peak around 50 kDa (**A**, left panel and **B**, center and right panels: compare grey and colored histograms). Such false positive peaks from molecules below the detection limit of mass photometry have been reported previously^73^. DNA in the presence of Co^2+^ bound to the glass slide, resulting in mass peaks centered around 60 kDa (**A**, middle panel and **B**, left and center panels: compare grey and colored histograms). The DNA sites used in these experiments are named above the figures, with sequences reported in Supplementary Table 5. The ligands used in each experiment are as per the labels within the figure panels. Source data

We confirmed that we obtained the intended stoichiometry of TW:DNA complexes using mass photometry. TW bound all single cognate binding sites in a 1:1 fashion (Extended Data Fig. 3a and Supplementary Table 1). Importantly, TW only formed 1:1 complexes with DNA containing two cognate sites in the presence of both corresponding ligands, even when using high nanomolar concentrations of DNA in 4–10-fold molar excess to TW (Fig. 3c, Extended Data Fig. 3b and Supplementary Table 1).

The timescales of sequential binding and release of the feet are crucial to achieving directional motion^19,27–29^. For TW, the time spent in a particular two-ligand solution must be short compared with the half-life of the corresponding two-foot-bound state, while the release of single feet on ligand removal must be fast compared with this timescale.

To determine these timescales, we studied the dissociation kinetics of TW:DNA complexes using an SPR displacement assay. During dissociation, a large excess of free, specific DNA was introduced to prevent TW from rebinding to surface-bound DNA, enabling the quantification of apparent off-rates (Fig. 3d, Extended Data Fig. 2, Extended Data Table 2 and Supplementary Discussion A). We found the half-lives for TW following the removal of controlling ligands to be <2 s. In the presence of single controlling ligands, the half-lives were 0.6–4 s. In both the absence and presence of controlling ligands, the MetJ foot was the fastest to dissociate from DNA, and the DtxR foot the slowest. The off-rates decreased dramatically in the presence of two controlling ligands, with half-lives of at least 30–50 s (Supplementary Discussion A). These measurements provided the boundaries for clocking TW to achieve processive stepping.

## Demonstrating TW stepping along a DNA track

**Fig. 4:**
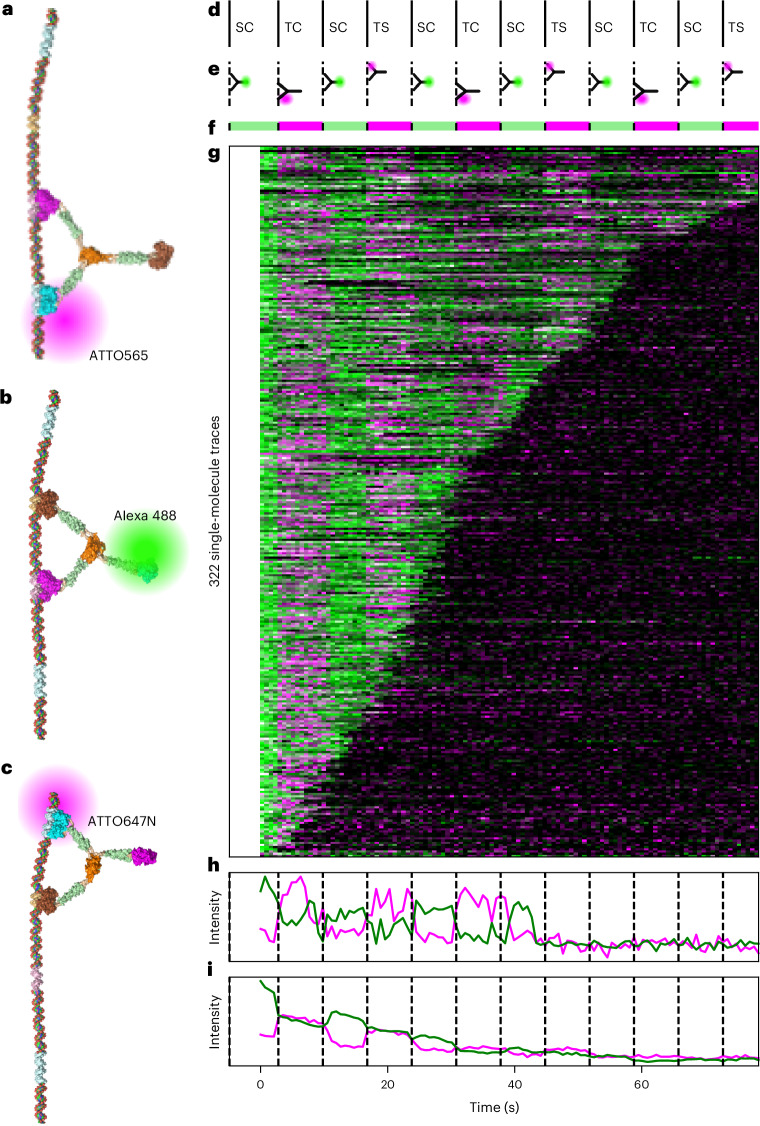
Single-molecule FRET Experiment 1: TW stepping on a DNA track in response to ligand changes. TW walking experiments were performed on a four-site (*trpR–dtxR–metJ–trpR* bottom to top) DNA track attached via biotin–streptavidin linker to a biotinylated PLL-*g*-PEG passivated surface. The *trpR* sites at the bottom and top were labelled with different FRET acceptors (ATTO565 and ATTO647N, respectively). The TrpR foot was labelled with the FRET donor Alexa Fluor 488, a green emitter. **a**–**c**, TW:DNA complexes that we expected to form in the presence of pairs of ligands: Trp + Co^2+^ in **a**, SAM + Co^2+^ in **b** and SAM + Trp in **c**. Each of the three complexes was designed to yield a distinct fluorescence signal on excitation with a 488 nm laser: FRET to ATTO565 (emission peak at 590 nm) when the TrpR foot is bound to the bottom-*trpR* site (**a**), Alexa Fluor 488 (emission peak at 525 nm) from the free TrpR foot (**b**) and FRET to ATTO647N (emission peak at 664 nm) when TrpR is bound to the top-*trpR* site (**c**). Two-colour imaging with emission split at 543 nm was used to simultaneously record Alexa Fluor 488 emission (shown in green) and ATTO565 plus ATTO647N emission (shown in magenta). **d**, The controlling solutions and how they are exchanged over time: Trp + Co^2+^ (TC), SAM + Co^2+^ (SC) and Trp + SAM (TS). **e**, Schematic representation of the intended position of the TW on the DNA track for the controlling solutions in **d**. **f**, The expected fluorescence signal from the TW:DNA complexes in **e**. **g**, Kymographs of all 322 detected co-localizing single molecules from a single field of view, sorted by time of last identified fluorescence. (**h**) Normalized example of single-molecule trace. (**i**) Sum of all single-molecule traces in the kymograph **g**. Source data

We examined TWs ability to walk along a DNA track using single-molecule Förster resonance energy transfer (smFRET). We attached a short DNA track containing four binding sites (bottom to top: *trpR*, *dtxR*, *metJ* and *trpR*) to a passivated surface (Fig. 4a–c). We labelled the track’s two *trpR* sites with different FRET acceptors, ATTO647N (emission peak at 664 nm) at the top and ATTO565 (emission peak at 590 nm) at the bottom. The corresponding FRET donor, Alexa Fluor 488 (emission peak at 525 nm), labelled the TrpR foot (Ser107; Supplementary Fig. 3 and Supplementary Tables 2 and 3), enabling FRET when the TrpR foot binds to either *trpR* site. This uniquely distinguished the three possible two-foot-bound states by their emission wavelengths: FRET emission peaking at 590 nm with TW bound to the bottom-*trpR* site and the *dtxR* site (Fig. 4a); emission peaking at 525 nm (that is, no FRET) with TW bound to the *dtxR* and *metJ* sites (Fig. 4b); and FRET emission peaking at 664 nm with TW bound to the *metJ* and top-*trpR* sites (Fig. 4c).

**Extended Data Fig. 4:**
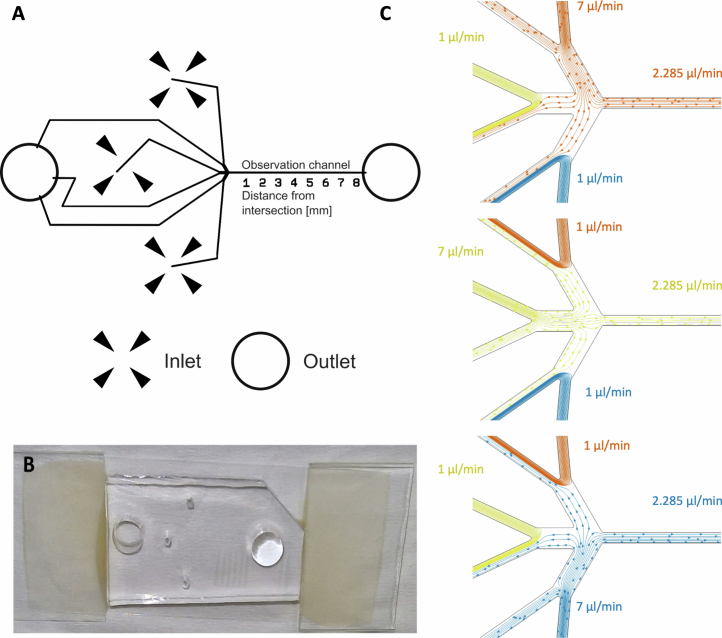
Design of microfluidic device for single-molecule tumbleweed stepping experiments. The device fulfills the key requirements for the control of TW: the fluid in the observation channel can be switched between three different ligand-pair solutions in arbitrary temporal order with a time resolution better than one second^30^. In our experiments, TW are localized in the observation channel approximately at position 1 mm from the intersection. (**A**) Locations where the three inlets are punched are marked with arrows pointing inwards. Each inlet is to be connected to a reservoir containing the running buffer with a pair of ligands. Each inlet is connected via an equal length escape channel to a common outlet (circle, far left). Directly following the intersection of the three inlet channels, the main observation channel can be seen labelled with distance in mm from the intersection. The observation channel terminates in another outlet (circle, far right) placed 10 mm from the intersection. (**B**) Photo of assembled microfluidic device bonded to a coverslip including glued glass slide fragments for support and mounting. (**C**) 2D finite element simulation (incompressible, laminar flow) of flow rates in the microfluidic device for three fluids (red, yellow and blue) injected into the device inlets, one at 7 µL/min (“on” channel) the other two at 1 µL/min (“off” channel). The fluid injected at 7 µL/min (Top: red, Middle: yellow, Bottom: blue) propagates in the observation channel at 2.285 µL/min while the other two fluids are directed to their respective “escape” channel.

To control TW stepping, we used a microfluidic device^30^ (Extended Data Fig. 4) that enabled switching between three control solutions in arbitrary temporal order, with a time resolution of less than 0.2 s (ref. ^30^). Ligand-pair solutions were changed at 7 s intervals, which was long enough to collect several fluorescence frames during each ligand step and to allow the slowest foot, DtxR, to dissociate (Extended Data Fig. 2 and Extended Data Table 2). Once one foot detached from the track, TW diffused within a volume around the remaining bound foot, enabling the next foot to bind to its cognate site on a sub-second timescale^28,29^. Given that two-foot-bound states have a half-lives of 30–50 s (Extended Data Fig. 2 and Extended Data Table 2), we expected to observe an average of >4 steps per TW.

**Fig. 5:**
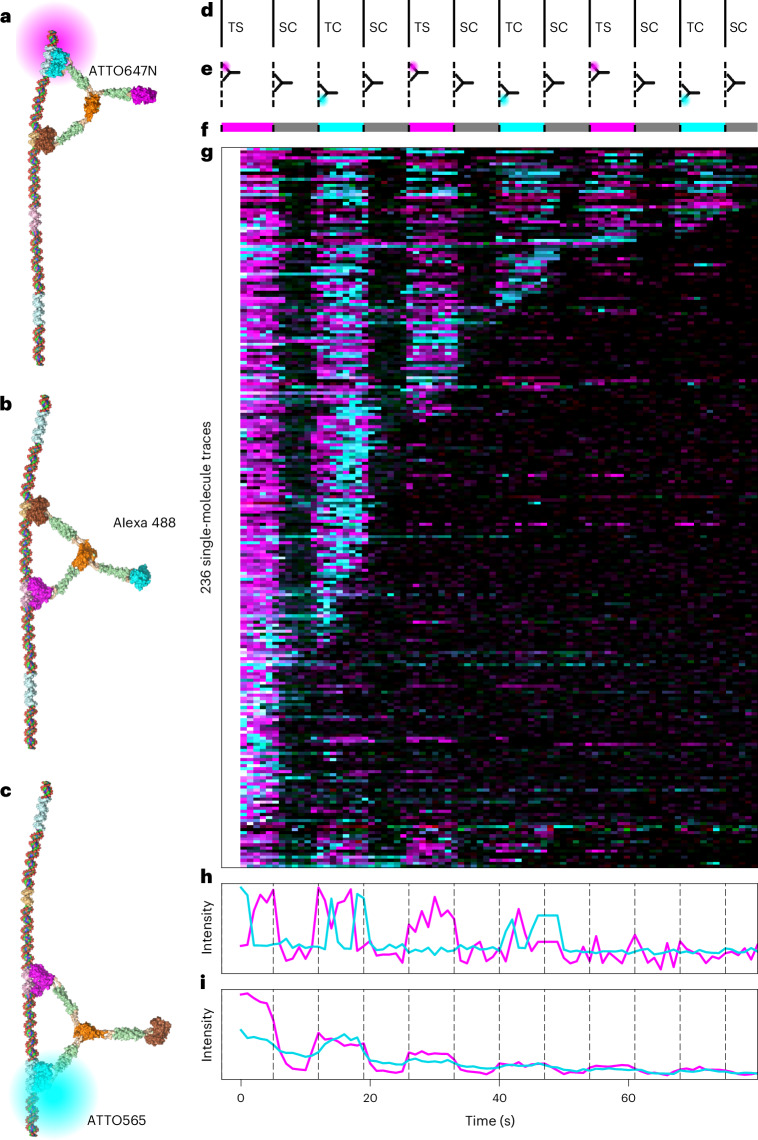
Single-molecule FRET Experiment 2: TW visits both ends of the DNA track. **a**–**c**, The same four-site DNA track and fluorescent labelling were used as in Fig. 4, but two-colour imaging was performed with emission split at 633 nm to simultaneously record ATTO565 emission and ATTO647N emission. In this way, we can distinguish whether the TW TrpR foot was bound to the top *trpR* site (**a**, FRET emission peak at 664 nm, shown in magenta) or the bottom *trpR* site (**c**, FRET emission peak at 590 nm, shown in cyan), whereas the centre position with a free TrpR foot (**b**) is expected to be recorded as dark. **d**, The controlling solutions and how they are exchanged over time: Trp + Co^2+^ (TC), SAM + Co^2+^ (SC) and Trp + SAM (TS). **e**, Schematic representation of the intended position of the TW on the DNA track at these times. **f**, The expected fluorescence signal from TW:DNA complexes for the states in **e**. **g**, Kymographs of all 236 detected co-localizing single molecules from four fields of view, sorted by time of last identified fluorescence. **h**, Example of a normalized single-molecule trace. **i**, Sum of all single-molecule traces in the kymograph **g**. Source data

We report two types of experiment (Figs. 4 and 5, respectively) that, together, fully characterize TW stepping up and down the four-site track. To generate stepping, we sequentially changed the ligand-pair solutions to induce TW to step down then up the track, repeating until no further stepping was observed (Figs. 4d,e and 5d,e). In each experiment, DNA:TW complexes were identified by co-localization, and time traces of background-subtracted fluorescence intensity from hundreds of individual complexes in a field of view were extracted.

Experiment 1 shows that the TrpR foot of TW binds and unbinds the *trpR* sites as the ligands are changed while TW remains attached to the track (Fig. 4d–i), thereby demonstrating that TW walks along the track. We added 1 nM TW to the fluidic device in the presence of Co^2+^ + SAM ligands to induce TW to bind to the central *dtxR* and *metJ* sites (Fig. 4b, e). We collected light simultaneously in two wavelength bands: the first suitable to observe emission peaking at 525 nm from the TW TrpR foot (green, Fig. 4b, e); and the second suitable for emission from both the 664 nm peak and 590 nm (magenta, Fig. 4a,c, e). This enabled us to observe both FRET positions (at the bottom and top of the track) in one channel while detecting the signal from the TW in the other. We observed fluorescence changes in synchrony with solution changes (Fig. 4d), with donor (green, Fig. 4g,h) and acceptor (magenta, Fig. 4g,h) signals alternating out of phase (Fig. 4h) until the signal was lost through bleaching or TW detachment. Within a single field of view, we identified 322 complexes showing 1:1 TW:DNA. Overlaid kymographs for the two observation channels (Fig. 4g) from this single field of view show the FRET state of all TW:DNA complexes. Nearly all alternate in synchrony with changes in the controlling solution, indicating stepping. The sum of these individual traces (Fig. 4i) further demonstrates the anti-correlated behaviour, in phase with changes in the controlling solution. Taken together, Experiment 1 confirms that the TrpR foot binds and unbinds to/from the *trpR* sites on the track as dictated by the ligands. However, it cannot discriminate between TrpR binding to the intended *trpR* site versus the *trpR* site at the opposite end of the track.

In Experiment 2 (Fig. 5), we confirmed that TW successfully visits both the top and bottom of the track in response to ligand control. We simultaneously imaged two-colour emission of the top and bottom FRET acceptors recorded in separate channels: TrpR foot bound to the top-*trpR* site (magenta, Fig. 5a,e); and TrpR foot bound to the bottom-*trpR* site (cyan, Fig. 5c,e). Examples of the anti-correlated FRET signals from the two *trpR* sites are shown in Fig. 5h and Supplementary Fig. 4. From overlaid kymographs from four separate fields of view (Fig. 5g), we found 236 1:1 TW:DNA complexes and nearly all exhibited dynamic behaviour correlated with ligand exchange. The dark bands coincide with periods where TW is bound to the central *metJ* and *dtxR* sites (Fig. 5b). The sum of the individual traces (Fig. 5i) shows that TW starts at the top *trpR* site, moves down through the dark central site and continues to step. The alternating dark and bright states confirm stepping, and the presence of magenta and cyan signals confirm that TW reaches both the top and the bottom of the tracks.

From individual traces (Fig. 5h and Supplementary Fig. 4), we found that, when the controlling solution contains Trp, single TWs have a highly dynamic behaviour, switching on a timescale of seconds between being bound to the top-*trpR* and the bottom-*trpR* sites (Fig. 5g,h and Supplementary Fig. 4). This indicates that TW can ‘overstep’, by binding to the non-adjacent *trpR* site. By analysing all traces during the first three solution conditions (Fig. 5g and Supplementary Fig. 4), we found that the TW TrpR foot bound the intended (adjacent) *trpR* site 65% ± 8% of the time (correct, directional stepping), whereas it bound the unintended, distal *trpR* site 35% ± 6% of the time (overstepping either forwards or backwards).

Control experiments show that no stepping by TW on the four-site track occurs when switching between solutions containing the same ligands (Supplementary Discussion B and Supplementary Figs. 5–7).

**Extended Data Fig. 5:**
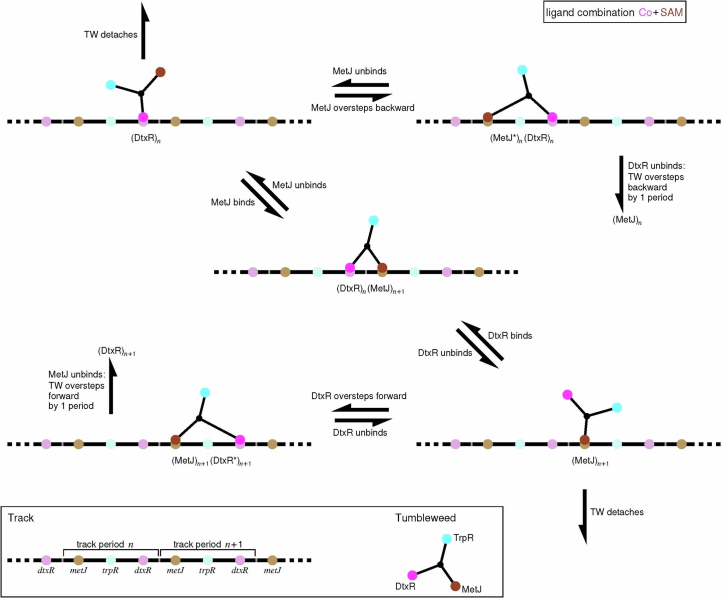
Illustration of states and transitions modelled by the rate equations. Lower panel: The DNA track, extending ad infinitum, is sketched by a horizontal black line with colored dots, which label the different cognate binding sites on the track (compare Fig. 1b in the main text); two track periods are marked as *n* and *n* + 1. The TW molecule is depicted in a star-like manner with three colored dots representing the repressor proteins TrpR (cyan), DtxR (magenta) and MetJ (brown), see Fig. 1 in the main text. Main panel: The five different possible states in the presence of the ligand combination Co^2+^ + SAM, including intended binding events and unintended overstepping events. The states are labelled by the repressor(s) bound to the track and the track period they are bound to, for example, (DtxR)_*n*_(MetJ)_*n*+1_ indicates that DtxR is bound to track period *n* and MetJ to track period *n* + 1 (this is the state in the middle); an asterisk at the repressor label indicates an overstepped configuration. The arrows indicate transitions between these different states. For the chosen ligand combination, there are no states in which TrpR is bound, or transitions towards such states, because we assume that TrpR can only bind in the presence of its ligand Trp. The sequence of events from the state (DtxR)_n_ (upper left corner) to the state (MetJ)_n+1_ (lower right corner) via the state (DtxR)_*n*_(MetJ)_*n*+1_ in the middle corresponds to the desired forward stepping behavior from one track period into the next. The transitions from the states (MetJ*)_*n*_(DtxR)_*n*_ and (MetJ)_*n*+1_(DtxR*)_*n*+1_, marked TW oversteps, end up in the states (MetJ)_n_ and (DtxR)_*n*+1_, which are equivalent to (MetJ)_n+1_ and (DtxR)_*n*_ but shifted by one track period backward and forward, respectively (the track:TW sketches of these shifted states are not included in the Figure).

**Extended Data Fig. 6:**
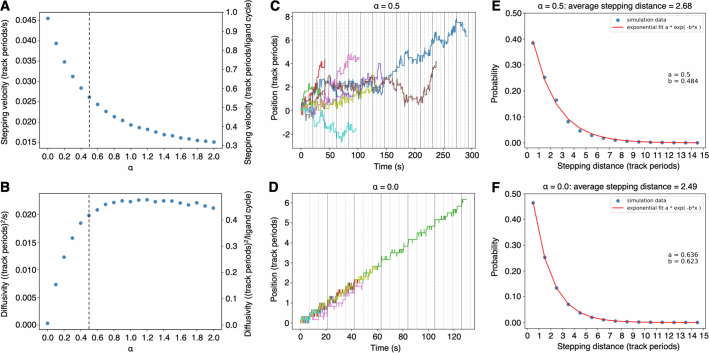
Simulations of tumbleweed walking along an infinite DNA track. (**A**, **B**) Dynamical characteristics of the stepping motion along the track as a function of the parameter α which controls the relative frequency of overstepping. For α = 0 there is no overstepping, α = 0.5 corresponds to the situation observed in experiments where 65% of the time TW correctly steps while 35% oversteps (vertical dashed lines), for α = 1 correct stepping and overstepping are equally likely, and for α > 1 overstepping dominates. (**A**) Average stepping velocity obtained from 500,000 independent realizations of the stepping simulation. For the units of velocity, a track period consists of three binding sites and a ligand cycle is considered a full cycle of three ligand solution changes, so that a velocity of one corresponds to ideal stepping. (**B**) Effective diffusion coefficient of the same set of 500,000 trajectories. (**C**, **D**) Trajectories of TW stepping along the DNA track from simulations. In each panel 10 independent simulation runs are shown in different colors. The vertical dashed lines indicate the time points at which the ligand combination in the solution is switched. (**C**) Trajectories with a probability of overstepping that reproduces the experimentally observed 65% correct stepping and 35% overstepping. (**D**) Trajectories without overstepping (only correct binding to the track is included in the model). Substeps visible in the traces correspond to binding/unbinding events within the current spatial period on the DNA track. (**E**, **F**) The distances the simulated TW steps along the track before detaching are exponentially distributed. From a fit to the exponential curves, we obtain the average distance covered before detaching from the track (in units of [track periods]). (**E**) α = 0.5 (experimentally observed frequency of overstepping); 1/e stepping distance is 2.1 track periods ≈ 103 nm. (**F**) α = 0 (no overstepping); 1/e stepping distance is 1.6 track periods ≈ 78 nm. Source data

**Extended Data Fig. 7:**
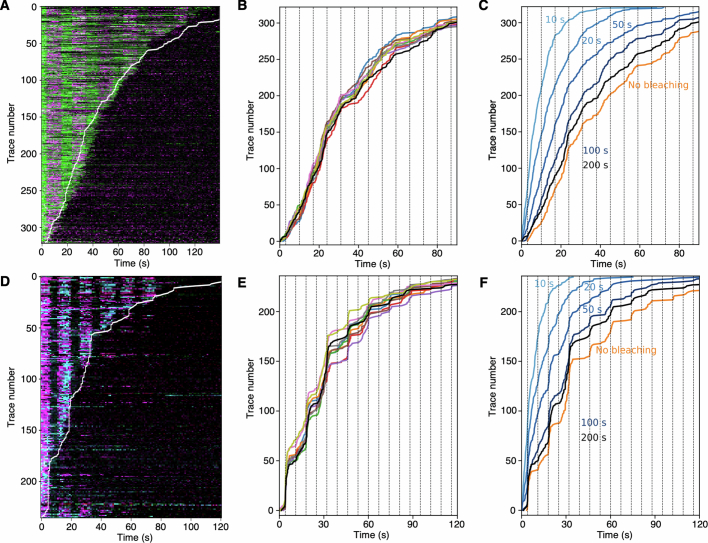
Simulation of single-molecule kymographs. Kymographs were simulated using the model described in Supplementary Discussion D. (**A–C**) Experiment 1 (c.f. Fig. 4g). (**A**) Overlay of simulated kymograph assuming a bleaching time of 200 s with the experimental kymograph from Fig. 4g. (**B**) 10 independent simulations using the same parameters as in Panel A demonstrating that the simulated kymograph in **A** (white curve) is typical. (**C**) Effect of varying the photobleaching time from 10 to 200 s. Orange curve shows the same simulation with no photobleaching. (**C–F**) Experiment 2 (c.f. Fig. 5g). **(D**) Overlay of simulated kymograph assuming a bleaching time of 200 s with the experimental kymograph from Fig. 5g. (**E**) 10 independent simulations using the same parameters as in Panel D demonstrating that the simulated kymograph in **D** (white curve) is typical. (**F**) Effect of varying the photobleaching time from 10 to 200 s. Orange curve shows the same simulation with no photobleaching. Source data

To understand how the observed stepping behaviour translates into TW’s ability to walk along an extended DNA track, we created a coarse-grained model based on Master equations governing the binding and unbinding events (Extended Data Figs. 5 and 6 and Supplementary Discussion C). On the basis of observed binding and unbinding rates (Extended Data Table 2) and the observed 35% likelihood to overstep (either forwards or backwards), the model reproduced the experimental kymographs when a bleaching time of 200 s was included (Extended Data Fig. 7 and Supplementary Discussion D). On an infinite track, the model predicts that TW would move directionally at a speed of about 0.5 track periods per full solution cycle (where a track period is 49 nm, the distance spanned by all three binding sites (Fig. 1b), and a solution cycle is the full cycle of three ligand changes; Extended Data Fig. 6a,b). For a 21 s experimental cycle time, this results in a speed of approximately 1.2 nm s^−1^. TW processivity with this cycle time is predicted to be 3–6 steps giving a run length of 50–100 nm (Extended Data Fig. 6c–f), which is governed by the observed detachment time in the presence of two ligands. Importantly, the model corroborates that on average TW directionality is robust to a wide range of overstepping probabilities (Extended Data Fig. 6).

## Conclusion

Our single-molecule experiments show that individual TWs successfully take up to 11 steps, controllably up and down a short track before dissociating. The observed overstepping likely occurs because of inherent flexibility of the DNA track (Supplementary Discussion E) and the TW linker regions. Dynamic, stochastic behaviour, including occasional mis-stepping, has been observed in single-molecule studies of biological motors, including myosin^31,32^, kinesin^33–36^ and dynein^37^ and it is predicted by stochastic thermodynamics as a fundamental characteristic of molecular motors^38–40^.

**Extended Data Fig. 8:**
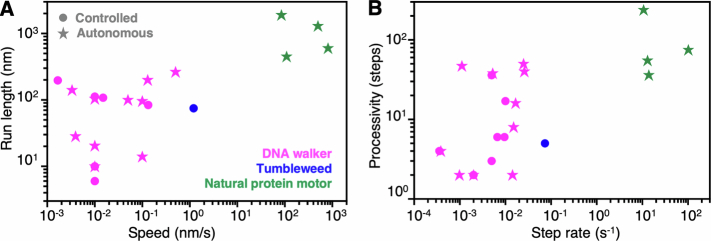
Comparison of tumbleweed performance to artificial DNA motors and natural protein motors. The comparison focuses on single-molecule constructs that perform directional motion. Large DNA nanoparticle and DNA origami burnt-bridge ratchets and non-directional diffusive walkers were excluded from the analysis. TW performance parameters were estimated from modelling in this manuscript (Extended Data Figs. 5–7 and Supplementary Discussions C and D). Artificial DNA motor performance parameters were taken from the references in Table 1 from the recent review Piranej et al. (2026)^13^, with natural protein motor parameters taken from the references in Box 1 of the same review. (**A**) Comparison of TW performance to artificial DNA walkers and natural protein motors in terms of run length (nanometers). (**B**) Comparison of TW performance to artificial DNA motors and natural protein motors in terms of processivity (steps). Source data

The main consequence of overstepping is variability in the displacement of individual TWs without introducing a directional bias (Extended Data Fig. 6), which is insensitive to the precise probability of overstepping over a wide parameter range (Extended Data Fig. 6). TW outperforms artificial DNA motor speed (typically 10^−2^–1 nm s^−1^) with comparable processivity^13^ (Extended Data Fig. 8). With a reduction in overstepping, which may be achievable by using DNA tracks based on rigid origami nanotubes^41^, our modelling predicts that TW approaches a speed of one track period per solution cycle. This corresponds to 2.3 nm s^−1^ using the 21 s experimental cycle time. While speed is also limited by the timescale for detachment of TW feet, a speed of 10–20 nm s^−1^ is likely achievable by decreasing the fluidic cycle time, bringing TW closer to that of natural protein motors (Extended Data Fig. 8).

By creating a protein walker from non-motor components, we have shown that a modular protein engineering approach can deliver an emergent, designed function—directional walking—that is beyond the sum of the components. This accomplishment reduces the size of synthetic protein-based motors from modular assemblies of tens to thousands of proteins^42,43^ to individual walker proteins constructed from modular domains. Our approach circumvents fundamental limitations in current protein design capabilities, namely the difficulty in creating proteins with the required dynamic properties for motor function, while mimicking how new functional proteins evolve in Nature by combining and assembling existing protein domains or protein fragments to render emergent properties^44^. Our proof-of-principle demonstration is evidence that complex, dynamic protein machines can be developed without requiring complete mastery of de novo design of flexible, information-transmitting protein structures.

Our clocked walker serves as an experimental platform for investigating the physical principles underlying nanoscale motor function, including the trade-offs between energy efficiency and performance predicted by stochastic thermodynamics^38–40^. It also establishes a critical foundation for developing fully autonomous, artificial motor proteins (see strategies for developing autonomous motor proteins^27,45^), where to date, successful engineered motor proteins have been chimeras, where the natural motor protein domains and functionality have been preserved^46,47^.

## Methods

### Construct design and cloning

Protein constructs were based on DtxR (residues 2–121 with a C102D mutation based on PDB 1F5T (ref. ^48^), UniProt Acc. No. P0DJL7), Geminin_Coiled-Coil_ (residues 110–145 with an additional N-terminal threonine and C-terminal glutamine based on PDB 1T6F (ref. ^49^), UniProt Acc. No. E2QRF9), TrpR (residues 2–10 based on PDB 1TRO (ref. ^50^), UniProt Acc. No. P0A881), MetJ (residues 2–105 with a Q45K mutation based on PDB 1MJM (refs. ^51,52^), UniProt Acc. No. P0A8U6) and the SpyTag:SpyCatcher system^53^.

Both MetJ and TrpR were expressed from pET28a vectors as fusion proteins with an N-terminal 8x His-tag. DtxR was expressed from a pET19b vector as a fusion protein with a C-terminal 6x His-tag.

TW was produced from two separate constructs, TW1 and TW2 (Fig. 1a and Supplementary Fig. 8). TW1 consists of DtxR-Geminin_Coiled-Coil_-SpyCatcher. TW2 consists of TrpR-Geminin_Coiled-Coil_-SpyTag-Geminin_CoiledCoil_-MetJ. For both TW1 and TW2, the domains were connected using glycine–serine-rich linkers. Both TW1 and TW2 constructs were synthesized by Genescript within a pET15b vector allowing protein expression with an N-terminal 8x His-tag and TEV protease cleavage site.

### Protein expression and purification

All proteins were expressed in *E. coli* (repressors and TW1 using BL21 in LB; TW2 using C41 in 2xTY media). Cells were grown at 37 °C until OD_600_ = 0.6, where temperature was reduced to 24 °C and expression induced using 0.4 mM IPTG. After 20 h, cells were collected by centrifugation and pellets stored at –80 °C.

Cell pellets were resuspended at 4 °C with stirring in 20 mM Tris pH 8, 150 mM NaCl, 20 mM imidazole, 10% glycerol, 2.5 mM MgCl_2_, 0.5 mM CaCl_2_, 1 mM NaN_3_, 0.1 mg ml^−1^ lysozyme, 10 µg ml^−1^ DNase I and 1 x protease tablet (Sigma-Aldrich). The slurry was lysed using a cell disruptor at 20 kPSI pressure. The soluble fraction was collected by centrifugation by 50,000 × *g* and loaded onto a 5 ml HisTrap HP nickel affinity chromatography column (Cytiva). The column was washed using combinations of buffer A (20 mM Tris pH 8, 400 mM NaCl, 10% glycerol, 1 mM NaN_3_) and buffer B (buffer A + 1 M imidazole): first with 2% buffer B, then 5% buffer B and then finally an elution step with 30% buffer B. Eluted proteins were concentrated and diluted to a final concentration of 3% buffer B, then subjected to an overnight TEV protease cleavage (1:50 mg mg^−1^ TEV:protein), with 1 mM DTT. The TEV-cleaved sample was then flowed over the HisTrap column to remove contaminants.

All proteins were subjected to SEC using either a HiLoad Superdex 75 26/60 column for individual repressors or a HiLoad 200 16/600 column for TW1 and TW2 (Cytiva). SEC was performed in 20 mM Tris pH 8, 400 mM NaCl, 10% glycerol, 1 mM NaN_3_ and 1 mM EDTA. Protein yields were typically 1 mg l^−1^ for individual repressors, 4 mg l^−1^ for TW1 and 0.3 mg l^−1^ for TW2. Purified protein was then frozen using liquid nitrogen and stored at –80 °C.

### Assembly and purification of TW

Purified TW1 and TW2 were mixed with a molar ratio of 1.2:1 (with TW2 at a final concentration of 20–40 µM) in 20 mM Tris pH 8, 400 mM NaCl, 10% glycerol, 1 mM NaN_3_ and 1 mM EDTA at 4 °C for 16 h. Assembled TW was purified by SEC using a Superdex 200 Increase 10/300 column (Cytiva), using assembly buffer. TW was then frozen in liquid nitrogen and stored at –80 °C.

### Fluorescent labelling of TW

Fluorescent TW was produced by thiol conjugation of a maleimide dye to TW containing a cysteine mutation. TW2 construct containing TrpR-S107C (TW2-S107C) was synthesized by Genscript. The protein was expressed and purified as described above, with 1 mM DTT included in purification buffers.

Alexa Fluor 488 C_5_ maleimide (AF488; ThermoFisher Scientific) was resuspended to 10 mM in DMSO. Purified TW2-S107C was reduced with 1 mM TCEP pH 7 before labelling at a final concentration of 30–60 µM with 5–10× molar excess of maleimide dye. Reactions were performed in 50 mM Tris pH 8, 400 mM NaCl and 10% (v/v) glycerol either for 2 h at room temperature or overnight at 4 °C. Reactions were quenched with 50 mM DTT for 30 min at room temperature. Labelled TW was assembled and purified as described above.

Following removal of excess dye, the degree of protein labelling was measured using an ND-1000 NanoDrop spectrophotometer (ThermoFisher Scientific). The absorbance of the protein:dye conjugate was measured at 280 nm (protein) and at 495 nm (AF488) (Supplementary Table 2). The degree of labelling was calculated according to the equation^54^

1 $${\mathrm{Degree}}\,{\mathrm{of}}\,{\mathrm{labelling}}=\frac{{\varepsilon }_{{\mathrm{Protein}}}\times {{\mathrm{A}}}_{495}}{({{\mathrm{A}}}_{280}{-{\mathrm{CF}}}_{280}{\times {\mathrm{A}}}_{495})\times {\varepsilon }_{{\mathrm{Dye}}}}.$$

Here ${\varepsilon }_{{\mathrm{Protein}}}$ is the extinction coefficient of the protein at 280 nm (55,350 M^−1^ cm^−1^), ${\varepsilon }_{{\mathrm{Dye}}}$ is the extinction coefficient of AF488 at 495 nm (73,000 M^−1^ cm^−1^) and CF_280_ is the correction factor for the absorption of light at 280 nm by AF488 (0.11). TW was assessed to be >95% labelled (two labels per dimeric TrpR foot), frozen with liquid nitrogen and stored at −80 °C.

### SAXS data collection

SAXS data were collected on the small/wide angle X-ray scattering beamline at the Australian Synchrotron^55^. The camera length was 2,680 mm corresponding to a *q*-range of 0.005–0.50 Å^−1^. Proteins were auto-loaded from a 96-well plate and 50 ml of 10 mg ml^−1^ of sample injected onto a Superdex 200 Increase 5/150 GL equilibrated in 10 mM HEPES pH 7.4, 150 mM NaCl and 5 mM MgCl_2_, connected in line with a coflow cell^56^ through which X-rays were passed at 8 × 10^12^ photons per second at 11.5 keV. The data was collected on a Pilatus 2M detector (Supplementary Table 4).

### SAXS data processing

SAXS data were processed using the ATSAS suite^57,58^ (Supplementary Table 4). Protein elution profiles were generated from scattering intensities using custom scripts. The elution profiles were used to determine the signals corresponding to protein and buffer frames. First, 21 buffer frames were selected and averaged for background subtraction. The radius of gyration (*R*_*g*_) was then determined for each frame across the protein peak (after buffer subtraction). Guinier analysis to determine *R*_*g*_ was performed in PRIMUS^57^ to monitor data quality. Distance distribution (*P*(*r*)) curves (Supplementary Fig. 1) were generated using GNOM^57^ to estimate the maximum dimension (*D*_max_) of the protein.

### Multi-state modelling with MultiFoXS

Multi-state modelling using MultiFoXS^24^ was used to determine the population-weighted conformational states of TW. The inputs to MultiFoXS included a TW model, SAXS profile, flexible hinge residues and rigid body connections. Models of TW1 and TW2 were generated using ColabFold v1.4: AlphaFold2 using MMseqs2^59^ and used to construct a complete model for TW assembly using UCSF Chimera^60^. For MultiFoXS, TW was divided into rigid elements and hinge regions. The rigid elements comprise three repressor dimers, three coiled-coils and SpyTag/SpyCatcher hub. Six hinges were placed in Gly-Ser-rich linkers. To simplify computation, the polypeptide chain was cut in one of the two Gly-Ser-rich linkers joining each rigid element. Thus, the six hinges were defined as TW1, Gly125-Gly126 and Gly170-Gly171, between DtxR, the coiled-coil and SpyCatcher, respectively; and TW2, Ser114-Gly115, Ser155-Ser156, Gly183-Ser184 and Gly228-Gly229, between TrpR, the coiled-coil, SpyTag, coiled-coil and MetJ, respectively (Supplementary Fig. 8).

Final calculations were performed to sample 10,000 conformations. For each sampled conformation, a SAXS profile is calculated followed by scoring of multi-state models to generate population-weighted ensembles with associated *R*_*g*_. The best multi-state models that fit the experimental SAXS data were based on the *χ*^2^ values and residual plots.

### Design and assembly of DNA tracks

The *metJ* cognate sequence (GAGACGTCTC) is the consensus Met box sequence from the MetJ:DNA crystal structure (PDB 1MJM)^51^. The *trpR* cognate sequence (GTACTCGCTAGCGAGTAC) was based on a *trpR*^*S*^ sequence that binds TrpR dimer with 1:1 stoichiometry^61^. The *dtxR* cognate sequence (TTAGGTTAACCTAA) was based on a DtxR:DNA crystal structure (PDB 1F5T)^48^, with the sequence altered to produce a palindrome that is capable of binding only one DtxR dimer.

The *metJ* flanking sequences were based on native *E. coli metC* protomer sequence (GenBank Ref. Seq. NC_000913.3) containing a single Met box^51^. The *dtxR* flanking sequences were conserved from the DtxR:DNA crystal structure (PDB 1F5T)^48^. The *trpR* flanking sequences were designed by a genetic algorithm to minimize the presence of cryptic repressor binding sites^26^. Double and quadruple site tracks were designed by arranging cognate sites and their flanking sequences. Models of the tracks were produced using cgNA+web^62,63^ and analysed using PyMOL^64^ to ensure that the cognate sites were appropriately separated and in phase.

All tracks were assembled from single strand oligonucleotides from Integrated DNA Technologies (IDT). Oligonucleotides were resuspended to 100 µM in Milli-Q water (Millipore) and stored at –20 °C.

Single- and double-site oligonucleotides were annealed at concentrations of up to 40 µM in 25 mM HEPES pH 7.4, 200 mM KCl, 5 mM MgCl_2_ in a thermocycler by heating the sample to 95 °C for 2 min followed by a 95–20 °C gradient at 1 °C min^−1^. Annealed tracks were stored at −20 °C. Supplementary Table 5 lists all DNA tracks used in SPR and mass photometry experiments.

Oligonucleotides for the four-site track used in single-molecule experiments are listed in Supplementary Table 6. Oligonucleotides required for the track were mixed at concentrations of 11.4 µM of each component in 1.25× T4 polynucleotide kinase (PNK) reaction buffer (New England BioLabs, catalogue number B0201S) and annealed as described above. The annealed four-site track was ligated by adding ATP (New England BioLabs, catalogue number P0756S) to a final concentration of 1 mM and T4 DNA ligase (New England BioLabs, catalogue number M0202S) to a final concentration of 10,000 U ml^−1^. The resulting ligation reaction mixture in 1× PNK was incubated at 16 °C for 16 h before heat inactivation at 65 °C for 10 min. DNA was purified using a spin column (QIAquick PCR Purification Kit, Qiagen) as per the manufacturer’s instructions. Purified tracks were stored at 4 °C.

### AFM

*E. coli* containing the pK8 plasmid^26^ were cultured in LB overnight in the presence of 25 mg ml^−1^ kanamycin, then pelleted by centrifugation for 3 min at 12,000 rpm and the plasmid purified using a MiniPrep kit (Qiagen). DNA was linearized by digestion with 1 U EagI per 150 ng DNA in NEBBuffer 3 for 2 h at 25 °C, followed by heat inactivation at 65 °C for 25 min. The product was purified using a PCR Cleanup kit (Qiagen) and digestion confirmed by gel electrophoresis.

TW (0.26 mg ml^−1^) and 10X HEPES buffer (40 mM HEPES, 100 mM NaCl, 20 mM MgCl_2_, pH 7.4) were both diluted fivefold in Milli-Q water (1:1:3 TW:10X HEPES:H_2_O). This stock (2.5 µl) was placed into a pre-lubricated microcentrifuge tube, to which was added 1.25 µl of linearized DNA (69.3 ng ml^−1^) and 1.25 µl of 40 mM tryptophan and incubated at room temperature on a spinner for 1 h. Afterwards, 45 µl of 1X HEPES buffer was added to the incubated solution, mixed and plated immediately onto freshly cleaved mica, where it was allowed to sit for 4 min. Excess solution was then shaken off, and the plate rinsed 5 times with 1 ml of Milli-Q water, then dried gently using filtered compressed air.

AFM images were recorded using an Asylum Research MFP-3D SPM, using Mikromasch HQ:NSC15/AL BS probes with a spring constant of 40 N m^−1^ and a resonance frequency of 325 kHz.

The chain-tracing software SmarTrace^65^ was used to determine the contour lengths of DNA flanking the bound TW proteins. For this analysis, *N* = 5 images were chosen that included seven or eight TW bound to clearly define the inserted track cassette. The cassette is located asymmetrically within the linearized plasmid, and thus the long and short contour lengths, and their ratio of lengths, confirmed that TWs were bound in the expected region of the DNA.

### SPR

SPR experiments were performed on a Biacore S200 instrument (Cytiva). All experiments were performed in running buffer (25 mM HEPES, pH 7.4, 200 mM KCl, 5 mM MgCl_2_, 0.2 mM EDTA, 0.05% (v/v) Tween-20), which was passed through a 0.22 µm filter and degassed by vacuum for at least 30 min. Data were collected at 40 Hz in multi-detection mode at 25 °C. All sensorgrams were double referenced.

DNA coating of SPR chips was prepared as previously described^66^. Series S CM5 sensor chips (Cytiva) were prepared by depositing approximately 5,000 RU of streptavidin onto the chip surface via amine coupling. A biotinylated single-stranded oligonucleotide (biotin anchor) was added to the chip for a response of 50–70 RU. Finally, annealed single or double cognate site DNA containing a single-stranded overhang complementary to the biotin anchor was flowed over the non-reference channels for a response of approximately 10 RU. Chips were regenerated using injections of 10 mM glycine pH 2.5, removing annealed DNA and while retaining the biotin anchor on the chip.

Individual repressors and TW were titrated onto single- and double-site DNA at 100 µl min^−1^. When required, ligands were used with the following concentrations: 1 mM SAM, 0.4 mM CoCl_2_ and 0.5 mM Trp. All titrations were performed using the A–B–A injection method, with a 30 s initial ligand injection, a contact injection containing protein for 15–30 s and a final ligand injection of 40–60 s to trigger dissociation. A 60 s injection of buffer containing 0.5 M KCl was used to remove any residual protein from the surface before the next titration point. The average steady-state positions were determined by the Biacore Evaluation software (Cytiva) and fit to a Hill equation using Prism 10 (GraphPad):

2 $${\mathrm{RU}}=\frac{{\mathrm{RU}}_{\mathrm{Max}}\times {\left[{\mathrm{Protein}}\right]}^{h}}{{{K}_{{\mathrm{d}}}}^{h}+{\left[\mathrm{Protein}\right]}^{h}}.$$

Here *K*_d_ is the apparent affinity, *h* is the Hill coefficient and RU_Max_ is the fitted maximum binding response.

We were unable to fit the above titrations to a simple 1:1 kinetic model. Increasing the amount of DNA on the chip surface leads to sensorgrams with slower apparent binding kinetics, indicating that the system suffered from mass transfer effects^67^. Protein:DNA interactions are particularly prone to mass transfer in SPR as electrostatically steered interactions lead to high association rates^68^. As a consequence, during the dissociation phase, the protein can release and rebind to the surface before it enters the bulk solution, resulting in a slower apparent dissociation rate^68,69^.

To avoid these complications, we studied TW:DNA dissociation kinetics using an SPR-based displacement assay based on classical tracer experiments^70,71^. A dual injection method was used to first load TW onto the surface DNA with an immediate second injection used to trigger dissociation. The assay was performed using a 100 µl min^−1^ flow rate. For single-site DNA experiments, a 5 nM TW solution was used to bind TW to DNA on the SPR chip for 25 s. Dissociation of the TW:DNA complex was triggered with an immediate 60 s injection of a solution containing 1 µM free DNA in solution ± ligand. The free DNA contained the relevant cognate binding site but lacked the anchor overhang to bind the sensor chip, hence preventing TW from rebinding chip-bound DNA. For experiments involving double-site DNA, 1 nM TW was loaded onto the DNA in the presence of two ligands for 60 s before dissociation was triggered with a 60 s injection of a solution containing both ligands and 1 μM of each of the relevant free DNA sequences. The resulting dissociation curves were fit to a single exponential decay function using Prism (GraphPad):

3 $${\mathrm{RU}}=\left({\mathrm{RU}}_{\mathrm{Initial}}-{\mathrm{RU}}_{\mathrm{NS}}\right)\times {{\mathrm{e}}}^{-{k}_{\mathrm{off}}\times t}+{\mathrm{RU}}_{\mathrm{NS}}.$$

Here RU_Initial_ is the initial response value, RU_NS_ is the non-specific response value, and *k*_off_ is the apparent dissociation rate constant. For double-site DNA, double-ligand experiments, RU_NS_ was set to 0.

### Mass photometry

Mass photometry experiments were performed as described^72^ on an Refeyn TwoMP instrument using 24 × 50 mm high-precision 1.5H coverslips (Marienfeld) and CultureWell gaskets (Grace Biolabs). Coverslips were prepared by 3 rounds of sonication in a 50% (w/w) isopropanol bath for 3 min followed by 3 min sonication in Milli-Q water, before being dried with nitrogen gas and stored at room temperature. Gaskets were affixed to the coverslip immediately before use.

Buffers (25 mM HEPES, pH 7.4, 200 mM KCl, 5 mM MgCl_2_, and 0.2 mM EDTA ± 0.5 mM SAM, 0.4 mM CoCl_2_ and/or 0.5 mM Trp) were passed through a 0.22 µm filter. Data were collected over a 60 s timeframe using the normal measurement mode and regular image size as set by the Refeyn AcquireMP software. Mass calibrations were performed using a combination of purified bovine serum albumin (Sigma-Aldrich) and NativeMark unstained protein standard ladder (ThermoFisher Scientific), with the 66 kDa, 132 kDa and 480 kDa peaks providing a calibration curve with *R*^2^ > 0.99. Manually picked mass distributions within the relevant mass ranges were selected and fit with a Gaussian function to determine an average molecular mass using the Refeyn DiscoverMP software. Signal interference by the ligand, SAM, resulted in a small peak around 50 kDa. Such false-positive peaks from molecules below the detection limit of mass photometry have been reported previously^73^. DNA transiently interacted with the glass surface in the presence of CoCl_2_, resulting in an additional mass peak centred around 60–80 kDa.

TW1, TW2 and TW samples were added to slides to a final concentration of 10 nM. TW and 4–10-fold molar excess DNA were preincubated for 5 min before analysis on the mass photometer. Final concentrations of 25–30 nM TW and 250–300 nM DNA were used for single-site DNA experiments, whereas double-site DNA experiments used 5–25 nM TW and 50–250 nM DNA.

### Microfluidics for single-molecule observation

To switch the TW running solutions, a microfluidic device, developed by Niman et al.^30^, was used (Extended Data Fig. 4). This device allowed switching between three solutions in arbitrary order, with sub-second time resolution, without stopping the flow. Flow rates were controlled using Fluigent lineup pumps, flow unit and OxyGEN software (Fluigent). Flow rates of 7 µl min^−1^ in the ‘on’ channel and 1 µl min^−1^ in the ‘off’ channels provided good switching (Extended Data Fig. 4c).

The chemical switching time where TW is located depends on the fluid speed and TWs location along the microfluidic channel^30^. Observations were conducted in the main observation channel, approximately 1 mm away from the intersection with inlet lines. A 3D finite element simulation (incompressible, laminar flow) of the volumetric flow rates in the device was performed using COMSOL Multiphysics software with the fluid set as water, with a density of 1,000 kg m^−^^3^ and a dynamic viscosity of 0.001 Pa s. For inlet flow rates of 7 µl min^−1^, 1 µl min^−1^ and 1 µl min^−1^, the simulated flow rate in the observation channel of 2.285 µl min^−1^ corresponded to a fluid speed of 7.6 mm s^−1^. On the basis of previous results^30^ (Extended Data Fig. 4), we expect that the chemical switching time in the experiments reported here at about 0.2 s or less, much faster than the 7 s during which each solution was held constant.

### Microfluidic master mould fabrication protocol

To make the master mould for the microfluidics, a photomask was fabricated using an MLA150 maskless lithography system (Heidelberg Instruments). A 4″ silicon wafer was dried at 180 °C for 5 min. A 50-µm-thick dry film resist (SUEX, K50, DJ Microlaminates) was applied using a laminating machine (Catena 35, Acco UK) at 65 °C. A pre-exposure bake was performed at 85 °C on a hotplate (Model 1000-1 Precision Hot Plate, Electronic Micro Systems) to remove air bubbles and to relax the film. The SUEX film was exposed at 365 nm in a contact mask aligner (Karl Suss MJB4 soft UV) for 27 s at a lamp power of 30 mW cm^−^^2^. Post exposure baking was done at 85 °C for 5 min. Development was done in Mr DEV 600 (Micro Resist Technology) for 15 min plus 5 min in fresh developer followed by rinsing in flowing isopropyl alcohol (IPA) and drying with nitrogen. A final bake was done in a convection oven at 200 °C for 15 min. To reduce adhesion of PDMS to the master, a layer of aluminium oxide (~1 nm) followed by a monolayer of perfluorodecyltrichlorosilane (FDTS) was deposited in an atomic layer deposition system (Fiji – Plasma Enhanced ALD, Veeco).

### Microfluidic device preparation and single-molecule observation

Coverslips (40 mm × 20 mm, #1.5 thickness, VWR) were prepared by sonicating in IPA (Merck) for 10 min and dried with pressurized nitrogen, followed by cleaning with piranha solution (3:1 sulfuric acid/hydrogen peroxide) for 30 min at 80 °C. Coverslips were then rinsed with Milli-Q water and dried with pressurized nitrogen. Final cleaning was performed in oxygen–nitrogen mixed plasma (Zepto Plasma Cleaner, Diener Electronic) for 3 min at 40 kHz, 100 W.

PDMS microfluidic devices were prepared by mixing a 2-component Sylgard 184 silicone elastomer and curing agent (G A Lindberg) in a 10:1 ratio and degassing to remove bubbles. The mixture was poured onto the master mould and cured at 80 °C for >1 h. After curing, using a 1 mm biopsy tool, inlets were punched at the three points indicated by inward pointing arrows (Extended Data Fig. 4a). Using a 3 mm biopsy tool, outlets were punched at the circles (Extended Data Fig. 4a), indicating the common waste outlet and the 10 mm mark along the primary observation channel. After washing with IPA, the moulded PDMS and coverslip contact surfaces were etched in nitrogen plasma for 10 s and bonded by placing the moulded PDMS onto the coverslip. Immediately, the device was wetted and then incubated for >1 h in imaging buffer (0.22 µm filtered 25 mM HEPES, pH 7, 200 mM NaCl, 5 mM MgCl_2_) with 1 mg ml^−1^ of 1:10,000 PLL-*g*-PEG-biotin (3.4 kDa PEG):PLL-*g*-PEG (2 kDa PEG). The device was visually inspected via white light transmission microscopy at 20× magnification for defects and then stored at room temperature in imaging buffer.

Before use, the devices were washed by aspirating from the common waste outlet and filling the observation channel outlet with imaging buffer. The device channels were then incubated in imaging buffer containing 20 nM streptavidin (Merck) for at least 10 min. Cut glass slides were glued to the ends of the coverslip using a two-component dental cement (Abberior Instruments) (Extended Data Fig. 4b). Immediately before imaging, 0.2 mg ml^−1^ TROLOX (Merck) was added to the imaging buffer, which was bubbled with nitrogen for 15 min to remove dissolved oxygen.

The microfluidic system was prepared by washing all microfluidic lines and flow meters with chlorine followed by Milli-Q water and then IPA followed by Milli-Q water. The cleaned microfluidic system was loaded with three 2 ml reservoirs of imaging buffer containing 2 mg ml^−1^ TROLOX (Merck) and either:

1. 0.5 mM Trp, 0.2 mM CoCl_2_
2. 0.2 mM CoCl_2_, 1 mM SAM
3. 1 mM SAM, 0.5 mM Trp.

For each control experiment, the microfluidic system was loaded with two reservoirs containing the same ligand-pair solution, and the third reservoir containing imaging buffer and 2 mg ml^−1^ TROLOX without ligands.

Finally, the device was connected to the microfluidic system via the three 1 mm inlets and rinsed for 1 min with running flows set to 7 µl min^−1^ for the solution desired in the observation channel and 1 µl min^−1^ for the two alternate solutions.

Single-molecule fluorescence imaging was performed using a Nikon-TI2 inverted optical microscope in TIRF mode. A 100× TIRF objective (Plan-APOCHROMAT 100× 1.45 NA Oil, Nikon) was used to collect fluorescence onto an sCMOS camera (Prime 95B, Photometrics), yielding a pixel size of 110 nm. Alternating laser excitation was provided by a laser combiner (LightHUB Ultra, Omicron) equipped with an acousto-optic tunable filter triggered by the camera digital trigger out via a multifunction I/O device (PCIe-6323, National Instruments). For excitation of Alexa Fluor 488, ATTO565 and ATTO647N laser excitation was alternated between 488 nm, 561 nm and 640 nm at intensities on the order of 100 W cm^−2^, 50 ms exposure time per excitation wavelength producing 3 camera frames with no time delay between wavelengths. A time sequence was generated by repeating this every 700 ms. Simultaneous imaging of two emission wavelength bands on two halves of the camera sensor was achieved using an image splitter (Optosplit II, Cairn). Two sets of filter combinations (Chroma) were used: for simultaneous imaging of Alexa Fluor 488 emission and combined ATTO565 and ATTO647N emission, a ZT543rdc dichroic with ET570LP and ET525/50m emission filters; and for simultaneous imaging of ATTO565 emission and ATTO647N emission, a ZT633rdc dichroic with ET600/50m and ET700/75m emission filters.

In situ on the microscope, both outlets were emptied by aspirating. A 200 µl mixture of 1 nM TW and 200 pM DNA track was prepared at room temperature and immediately pipetted into the observation channel outlet. All flows were set to 0.5 µl min^−1^ and a rolled kimwipe tissue paper (Kimtech) inserted into the common waste outlet to wick away waste buffer. The surface density of reagents was observed in the observation channel approximately 1 mm away from the intersection. Once single molecule binding on the surface began to reach approximately 1 µm^−2^, the flows were set to operating values of 7 µl min^−1^ and 1 µl min^−1^, and the tissue was removed. An unexposed field of view was selected, and the experiment was started. During the experiment, the solution present in the main observation channel was driven with 7 µl min^−1^, and the alternate solutions at 1 µl min^−1^. Solutions were changed every 7 s following the patterns indicated in Figs. 4d–f and 5d–f and Supplementary Figs. 5c, 6c and 7c.

### Single-molecule FRET analysis

The pixel coordinates of bright spots (Supplementary Fig. 9), independent of time point and colour channel, were found using the scikit-image package^74^. For each colour channel, a maximum intensity, *I*, projection over time was calculated and normalized with

4 $${I}_{\mathrm{norm}}=\frac{I-\min \left(I\right)}{\max \left(I-\min \left(I\right)\right)}.$$

From this image, a scale-space volume is generated by computing the Laplacian of Gaussian filtered image with successively increasing standard deviation and generating a stack of images from the result. Single particles were detected using the local maxima in this scale-space volume^75^. Detections close to the edges (35 pixels) of the field of view were removed.

The list of detected coordinate centres was filtered to include only detections that exhibited co-localization between the TW donor and DNA acceptor FRET channels (Supplementary Fig. 9). First, the nearest-neighbour pairs with one member in the donor channel and one in the FRET channel were identified. Co-localizing detections were determined as nearest neighbours closer than 330 nm (3 pixels) of each other to allow for localization uncertainty, alignment of the optosplit and chromatic aberration.

For each identified co-localization, a background subtracted intensity trace over time was extracted from the full unnormalized image sequence by calculating each frame as the average intensity of the pixels within a radius of 3 pixels (Supplementary Fig. 10a) minus the median of the intensities in the pixels a radius of 4 pixels away (Supplementary Fig. 10b). Doing this for each frame and channel resulted in the traces shown in Supplementary Fig. 11. These traces were matched to the ligand solution in the observation channel at the time of the image acquisition by comparing start elapsed times from the pump log and the image metadata. Any trace where the intensity of the first frame was either 2.5 times greater than or 0.2 times less than the average single fluorophore intensity was considered a spurious detection (for example, aggregates or unidentified debris) and removed from the analysis. A total of 1,860 particles were detected in Experiment 1, and 2,678 in Experiment 2. Out of these, 440 and 322, respectively, were determined to co-localize with DNA tracks. From these, 322 and 236 traces (Figs. 4g and 5g, respectively) were determined to be 1:1 TW:DNA complexes based on their fluorescence intensity.

Traces were sorted based on the last detected falling edge in either colour channel. Edges (Supplementary Fig. 12a) were determined by Gaussian filtering with a standard deviation of 1.2 (Supplementary Fig. 12b) and taking the first derivative (Supplementary Fig. 12c). Peaks and troughs were identified using sci-pys peakfinder^76^ using a threshold of 0.3 (Supplementary Fig. 12c). Each trace was then normalized between its max value as 1 and the average value of the final 15 frames of the trace as 0. Normalized traces were mapped to colours. In Experiment 1, 24-bit RGB colour mapping was used with acceptor intensity represented by magenta and donor intensity by green (Fig. 4g). In Experiment 2, CMYK colour space mapping was used with ATTO647N acceptor intensity represented by magenta and ATTO565 acceptor intensity represented by cyan (Fig. 5g).

Thresholding of the normalized intensity traces was used to determine the time which TW spent either bound to the intended (adjacent) *trpR* site or bound to the non-adjacent *trpR* site farthest from the bound foot. Owing to the differing FRET efficiencies of the two fluorophores, independently selected absolute thresholds were used, specifically 0.8 for ATTO647N FRET and 0.5 for ATTO565 FRET. Frames were counted as intended binding if the only the corresponding intensity exceeded the threshold: ATTO647N in the first buffer and ATTO565 in the third. Otherwise, frames were counted as non-adjacent binding including frames in which neither or both FRET intensities exceeded the threshold. To include an equal amount of data from each ligand solution, only the first three solution conditions were included in the analysis and only traces where the protein remained co-localized with the DNA for the full duration of this period were included. The fraction of time spent in the intended binding sites was calculated as ${t}_{\mathrm{intended}}/({t}_{{\mathrm{intended}}}+{t}_{{\mathrm{unintended}}})$ for each trace resulting in a mean of 65% ± 7.7%, while the fraction of time spent in the unintended binding sites was calculated as ${t}_{{{\mathrm{unintended}}}}/({t}_{{{\mathrm{intended}}}}+{t}_{{{\mathrm{unintended}}}})$ for each trace resulting in a mean of 35% ± 6.3% where limits are 95% confidence intervals.

### Statistics and reproducibility

All experiments were conducted with at least three independent replicates. No statistical method was used to predetermine sample size. The experiments were not randomized and the investigators were not blinded to allocation during experiments and outcome assessment.

## Online content

Any methods, additional references, Nature Portfolio reporting summaries, source data, extended data, supplementary information, acknowledgements, peer review information; details of author contributions and competing interests; and statements of data and code availability are available at 10.1038/s41565-026-02211-3.

## Supplementary information

Supplementary Information Supplementary Discussions A–E, Tables 1–6 and Figs. 1–13.

Supplementary Data 1 Source data for Supplementary mass photometry, SAXS and SPR data—Supplementary Table 1 and Supplementary Figs. 1 and 2.

Supplementary Data 2 Source data for Supplementary single-molecule data—Supplementary Figs. 4–7 and 9–12.

## Source data

Source Data Fig. 2 Excel spreadsheet of molecular masses, SAXS and Guinier plot; unprocessed SDS–PAGE image, mass photometry data output and AFM image.

Source Data Fig. 3 Excel spreadsheet of SPR and mass photometry data; mass photometry data output.

Source Data Fig. 4 .csv files of single-molecule FRET data.

Source Data Fig. 5 .csv files of single-molecule FRET data.

Source Data Extended Data Table 1 Excel spreadsheet of SPR affinity parameters.

Source Data Extended Data Table 2 Excel spreadsheet of SPR dissociation kinetics parameters.

Source Data Extended Data Fig. 1 Excel spreadsheet of SPR titration data.

Source Data Extended Data Fig. 2 Excel spreadsheet of SPR dissociation data.

Source Data Extended Data Fig. 3 Mass photometry output data.

Source Data Extended Data Fig. 6 Text files of simulation output.

Source Data Extended Data Fig. 7 Text files of single-molecule FRET and simulation output.

Source Data Extended Data Fig. 8 Excel spreadsheet of the speed and processivities of molecular motor.

## Data Availability

SAXS data have been deposited with the Small Angle Scattering Biological Data Bank (SASBDB) with the accession code SASDWM8. All other data are available in the main text or the supplementary materials. Source data are provided with this paper.

## Extended data

**Extended Data Table 1:** Tumbleweed:DNA affinities in the presence of a single ligand by SPR

DNA	Protein	Ligand	Apparent *K*_*d*_ (nM)	h	n
*metJ*	MetJ	SAM	4 ± 0.4	1.22 ± 0.02	4
*metJ*	TW	SAM	18 ± 5	0.96 ± 0.07	4
*dtxR*	DtxR	Co^2+^	5.5 ± 0.2	0.99 ± 0.03	5
*dtxR*	TW	Co^2+^	8 ± 1	0.92 ± 0.10	4
*trpR*	TrpR	Trp	48 ± 2	1.61 ± 0.02	4
*trpR*	TW	Trp	27 ± 4	1.07 ± 0.03	4
*trpR-dtxR*	TW	Co^2+^	2 ± 1	1.1 ± 0.1	4
*trpR-dtxR*	TW	Trp	15 ± 4	1.27 ± 0.09	4
*dtxR-metJ*	TW	SAM	14 ± 2	1.2 ± 0.2	3
*dtxR-metJ*	TW	Co^2+^	5 ± 2	1.2 ± 0.1	3
*dtxR-metJ*	TW	Trp	50 ± 20	1.72 ± 0.03	3
*metJ-trpR*	TW	SAM	18 ± 2	1.3 ± 0.2	3
*metJ-trpR*	TW	Trp	13 ± 4	1.22 ± 0.09	4

Affinities were determined by titrating TW onto a surface containing DNA. The average steady state response units in the contact phase were fitted to the Hill equation to determine the apparent *K*_*d*_ and Hill coefficient (h). Values represent average ± SEM, calculated from replicate experiments with sample size of n.

Source data

**Extended Data Table 2:** Tumbleweed:DNA binding kinetics by experiment and simulation

		Experiment			Simulation	
DNA	Ligand	k_off_ (s^−1^)	Half-life (s)	n	k_off_ (s^−1^)	k_on_ (s^−1^)
*metJ*	No ligand	3.7 ± 0.3	0.19 ± 0.02	3	3.7	0
	SAM	1.14 ± 0.06	0.61 ± 0.03	3	1.14	10
*dtxR*	No ligand	0.45 ± 0.02	1.56 ± 0.07	3	0.45	0
	Co^2+^	0.164 ± 0.009	4.2 ± 0.4	3	0.164	10
*trpR*	No ligand	0.99 ± 0.02	0.70 ± 0.02	3	0.99	0
	Trp	0.36 ± 0.04	2.0 ± 0.2	3	0.36	10
*trpR-dtxR*	Trp + Co^2+^	0.0239 ± 0.0004	29 ± 0.5	3	0.011	n/a
*dtxR-metJ*	SAM + Co^2+^	0.0152 ± 0.0007	46 ± 2	3	0.031	n/a
*metJ-trpR*	SAM + Trp	0.0233 ± 0.0001	29.7 ± 0.2	3	0.067	n/a

Apparent dissociation kinetics were determined experimentally using SPR. TW was loaded onto a DNA surface in the presence of the relevant ligand(s). Dissociation was triggered by injecting a solution containing no TW, the relevant ligand(s), and 1 µM DNA. 1 µM of each of the relevant single site DNA oligonucleotides were used for double ligand dissociation experiments. Dissociation curves were fitted to a single exponential decay function to determine apparent k_off_. Values represent average ± SEM, calculated from replicate experiments with sample size of n. Half-lives were calculated from the apparent k_off_ values. Simulated rates for TW:DNA binding are also reported. Single- and no-ligand off-rates were set as equal to the SPR dissociation experiments. The on-rates are assumed to be repressor-independent and are chosen to reproduce the measured double-ligand rates as well as possible; the double-ligand rates resulting from the model are all within a factor 3 from the measured rates. They are extracted from the model by simulating the detachment of TW from the track in the presence of fixed ligand combinations Trp + Co^2+^, Co^2+^ + SAM, and SAM + Trp, respectively. Transitions between unbound state and overstepped bound state use the same off-rates, but all on-rates are rescaled by a factor α.

Source data

## Code availability

The code used for single-molecule analysis is available via Zenodo at 10.5281/zenodo.20273367 (ref. ^77^). The code for the coarse-grained TW model is available via Zenodo at 10.5281/zenodo.20283947 (ref. ^78^). The code for SmarTrace to measure DNA contour lengths is available via GitHub at https://github.com/FordeLab/SmarTrace.
