# Supplementary material for: Clocked stepping of an artificial protein walker along a DNA track
Source: Nat Nanotechnol. 2026 Jul 6;21(7):987–95. doi: 10.1038/s41565-026-02211-3 (PMC13379313; doi:10.1038/s41565-026-02211-3)
Supplement: Supplementary file 1 — Supplementary Discussions A–E, Tables 1–6 and Figs. 1–13. [file 41565_2026_2211_MOESM1_ESM.pdf]

nature nanotechnology

Supplementary information https://doi.org/10.1038/s41565-026-02211-3

Clocked stepping of an artificial protein walker along a DNA track

In the format provided by the authors and unedited

Protein	Ligand	DNA	Theoretical Mass (kDa)	Observed Mass (kDa)	n
TW1	-	-	59	56 ± 2	4
TW2	-	-	74	72 ± 3	4
TW	-	-	133	133 ± 1	4
TW	-	*metJ*	133	133 ± 3	3
TW	SAM	*metJ*	155	150 ± 1	3
TW	-	*dtxR*	133	133 ± 2	3
TW	Co^{2+}	*dtxR*	159	154 ± 1	3
TW	-	*trpR*	133	132 ± 1	3
TW	Trp	*trpR*	161	150 ± 2	4
TW	-	*trpR-dtxR*	133	129 ± 2	3
TW	Trp	*trpR-dtxR*	188	181.7 ± 0.7	3
TW	Trp + Co^{2+}	*trpR-dtxR*	188	187 ± 2	4
TW	-	*dtxR-metJ*	133	130 ± 3	3
TW	Trp	*dtxR-metJ*	315 (2TW+DNA)	316 ± 3	4
TW	Co^{2+} + SAM	*dtxR-metJ*	182	184 ± 2	5
TW	-	*metJ-trpR*	133	128 ± 2	3
TW	Trp	*metJ-trpR*	183	178 ± 2	3
TW	SAM + Trp	*metJ-trpR*	183	183 ± 3	4

Supplementary Table 1. TW construct and complex molecular masses. Protein and protein:DNA molecular masses were determined using mass photometry, as shown in Fig. 3C & Extended Data Fig. 3. Theoretical masses were calculated assuming TW and TW constructs act as obligate dimers. Observed masses represent average ± SEM, calculated from replicate experiments with sample size of n. TW formed a complex with the *dtxR-metJ* track in the presence of Trp that was consistent with a 2:1 TW:DNA complex (theoretical mass 315 kDa).

Dye	A_{max} (nm)	Extinction Coefficient ($M^{-1}cm^{-1}$)	Correction Factor	Molecular Weight (g/mol)
Alexa Fluor™ 488 C_5 Maleimide	495	73000	0.11	720.66
Alexa Fluor™ 647 C_2 Maleimide	650	265000	0.03	1250
ATTO647N Maleimide	647	120000	0.04	829

Supplementary Table 2. Properties of dye conjugates used for protein labelling. Data from ThermoFisher Scientific and Sigma Aldrich.

**DNA acceptor**	**EC (m^{-1} cm^{-1})**	**$QY_{Acceptor}$**	**J(λ) ($x10^{15}$ M^{-1} cm^{-1} nm^4)**	**R_0 (Å)**	**R_0 x $QY_{Acceptor}$**	**Donor–Acceptor distances (Å)**
ATTO565	120000	0.9	3.99	64.01	57.61	38.0 & 44.1
ATTO647N	150000	0.65	0.94	50.33	32.72	32.9 & 46.3

Supplementary Table 3: TW-TrpR-S107C and track FRET properties. Information about quantum yield of the acceptor dye (QY), Extinction coefficient (EC), overlap integral (J(λ)) and Förster distance (R_0) were taken from https://www.fpbase.org/fret/. Values assume the refractive index $n = 1.33$ and the orientation factor $\kappa^2 = 2/3$. TW-TrpR-S107C was labelled with the donor fluorophore Alexa Fluor 488 (QY = 0.92). smFRET between Alexa Fluor488 and ATTO647N has previously been validated[1,2]. Donor–Acceptor distances were calculated by measuring the distance between the Cα of TrpR S107 (PDB: 1TRO) to the phosphate backbone of a DNA model generated by cgNA+ web[3] (Supplementary Fig. 3). Two distances for each donor–acceptor pair are reported as TrpR could bind to either strand of its palindromic DNA binding site.

(a) Sample details	
Organism	Synthetic construct
Source	Recombinant expression of components TW1 and TW2 in E. coli.
Scattering protein composition	TW protein composed of constructs TW1 and TW2 linked covalently via SpyTag/SpyCatcher
Sample environment/configuration	
Solvent composition	10 mM HEPES pH 7.4, 150 mM NaCl, 5mM $MgCl_2$
Sample temperature (°C)	Sample stored at 10°C in a 96-well plate, but measured at 20°C
In-beam sample cell	Sheath flow configuration
SEC-SAXS measurements	
Initial sample concentration (mg/mL)	10
Column type	Superdex 200 Increase 5/150 GL
Volume injected (μL)	50
Flow rate (mL/min)	0.2

(b) SAS data collection	
Data acquisition / reduction software	SAXS/WAXS beamline control software, *ScatterBrain* (v 2.30), *CHROMIX* [4]
Source/instrument description	SAXS/WAXS beamline at the Australian Synchrotron, with Pilatus 2M detector at a SDD of 2680mm, with an incident intensity of 8 x 10^{12} ph/s
Measured q-range (q_{min}–q_{max}) (Å^{-1})	0.005 – 0.5
Method for scaling intensities	Absolute scaling (cm^{-1}) referenced to water
Exposure time(s), No. of exposures	1 s x 21 frames for buffer/background<br>1 x 10 frames for sample
Additional relevant details	Buffer scattering frames taken immediately before the void fraction. Sample scattering frames taken at the elution peak over a region of stable R_g

(c) SAS-derived structural parameters	*PRIMUS/qt*, *AUTORG* and *GNOM* [4]
Guinier analysis	
$I(0) \pm \sigma$ (cm^{-1})	0.06175 ± 0.00022
$R_g \pm \sigma$(Å)	67.7 ± 0.6
qR_g range (datapoint range)	0.39 – 1.00 (points 4-30)
Linear fit assessment (*AUTORG* fidelity)	0.47
PDDF/$P(r)$ analysis	
$I(0) \pm \sigma$ (cm^{-1})	0.06177 ± 0.00015
$R_g \pm \sigma$ (Å)	69.1 ± 0.4
d_{max} (Å)	270
q-range (Å^{-1})	0.006 – 0.492
$P(r)$ reciprocal-space fit: χ^2, CorMap P-value	1.63, 0.106

(*d*) Scattering particle size.	
Method(s)/software	*MoW* from *PRIMUS/qt* [4]

Volume estimates (Å^3)	
Porod volume V_P	190
Molecular mass M estimates (kDa)	
Molecular mass M estimates	156
From chemical composition	133

(*e*) Modelling.	
Methods/software	
Atomistic modelling/software	*Multi-FoXS* [5]
q-range for fit (Å^{-1})	0.006 – 0.492
Symmetry/anisotropy assumptions	*N/A*
No. of individual model reconstructions	2 structures used to represent the scattering data taken from a pool of 100 conformations
χ^2, CorMap P-value	1.56, 0.156

(*f*) Data and model deposition.	
SASBDB ID	SASDWM8

Supplementary Table 4. SEC-SAXS data collection, reduction and analysis.

Oligo name	Oligo type	Oligo sequence
NR019_Biotin-Anchor	Anchor	ACCTTAGGGCGTGG-Biotin
NR139_metJ_top	Single site	CCACGCCCTAAGGTATGCTAGTTT**GAGACGTCTC**AAAACAGGA
NR140_metJ_bottom	Single site	TCCTGTTTT**GAGACGTCTC**AAACTAGCAT
NR141_dtxR_top	Single site	CCACGCCCTAAGGTCATGCAAGGC**TTAGGTTAACCTAA**CTTGCATGTT
NR142_dtxR_bottom	Single site	AACATGCAAG**TTAGGTTAACCTAA**GCCTTGCATG
NR206_trpR_top	Single site	CCACGCCCTAAGGTGAGCATAGTG**GTACTCGCTAGCGAGTAC**CTCTGACTTC
NR207_trpR_bottom	Single site	GAAGTCAGAG**GTACTCGCTAGCGAGTAC**CACTATGCTC
NR349_metJ_top	Competing DNA	GTTT**GAGACGTCTC**AAAA
NR350_metJ_bottom	Competing DNA	TTTT**GAGACGTCTC**AAAC
NR351_dtxR_top	Competing DNA	AGGC**TTAGGTTAACCTAA**CTTG
NR352_dtxR_bottom	Competing DNA	CAAG**TTAGGTTAACCTAA**GCCT
NR353_trpR_top	Competing DNA	AGTG**GTACTCGCTAGCGAGTAC**CTCT
NR354_trpR_bottom	Competing DNA	AGAG**GTACTCGCTAGCGAGTAC**CACT
NR420_trpR-dtxR_top	Double site	CCACGCCCTAAGGTGAGCATAGTG**GTACTCGCTAGCGAGTAC**CTCTGACTTCCATGACGGGTGTCACATCATGCAAGGC**TTAGGTTAACCTAA**CTTGCATGTT
NR421_trpR-dtxR_bottom	Double site	AACATGCAAG**TTAGGTTAACCTAA**GCCTTGCATGATGTGACACCCGTCATGGAAGTCAGAG**GTACTCGCTAGCGAGTAC**CACTATGCTC
NR423_dtxR-metJ_top	Double site	CCACGCCCTAAGGTCATGCAAGGC**TTAGGTTAACCTAA**CTTGCATGTTGATCATATAGCCAATATGCTAGTTT**GAGACGTCTC**AAAACAGGA
NR424_dtxR-metJ_bottom	Double site	TCCTGTTTT**GAGACGTCTC**AAACTAGCATATTGGCTATATGATCAACATGCAAG**TTAGGTTAACCTAA**GCCTTGCATG
NR426_metJ-trpR_top	Double site	CCACGCCCTAAGGTATGCTAGTTT**GAGACGTCTC**AAAACAGGAATGGCACGCGTCCTGAGCATAGTG**GTACTCGCTAGCGAGTAC**CTCTGACTTC

NR427_metJ-trpR_bottom	Double site	GAAGTCAGAG**GTACTCGCTAGCGAGTAC**CACTATGCTCAGGACGCGTGCCATTCCTGTTTT**GAGACGTCTC**AAACTAGCAT

Supplementary Table 5. Oligonucleotide sequences used for SPR and mass photometry. Underlined sequences represent complements to Biotin-Anchor strand used to anneal DNA surfaces in SPR experiments. Single- and double-site oligos refer to sequences that contain one and two repressor cognate sites, respectively. Competing DNA refers to sequences containing a single cognate binding site that was used to trigger dissociation in SPR experiments. Bold sequences represent the cognate binding sequences for the relevant repressor. Top and bottom sequences were annealed to form double-stranded DNA for use in experiments.

Oligo name	Description	Sequence
Up-strand 1	Anchor + bottom-*trpR* site + *dtxR* site	CCACGCCCTAAGGTGAGCATAGTG**GTACTCGCTAGCGAGTAC**CTCTGACTTCCATACGGGTGTCACATCATGCAAGGC**TTAGGTTAACCTAA**C
Up-strand 2	*metJ* site + top-*trpR* site	Phosphate-TTGCATGTTGATCATATAGCCAATATGCTAGTTT**GAGACGTCTC**AAAACAGGAATGGCACGCGTCCTGAGCATAGTG**GTACTCGCTAGCGAGTAC**CTCT
Down-strand 1	ATTO647N + top-*trpR* site 2	ATTO647N-AGAG**GTACTCGCTAGCGAGTAC**CACTATGCTCAGGACGCGTGCCATT
Down-strand 2	*metJ* site + *dtxR* site	Phosphate-CCTGTTTT**GAGACGTCTC**AAACTAGCATATTGGCTATATGATCAACATGCAAG**TTAGGTTAACCTAA**GCCTTGCATG
Down-strand 3	bottom-*trpR* site + ATTO565	Phosphate-ATGTGACACCCGTATGGAAGTCAGAG**GTACTCGCTAGCGAGTAC**CAC-ATTO565
Down-strand 4	Anchor	TATGCTCACCTTAGGGCGTGG-Biotin

Supplementary Table 6. Oligonucleotide sequences used to construct the track for single-molecule FRET. The four-site track was annealed, ligated, and purified as described in Methods from the six oligonucleotides listed here. Up-strands refer to strands designed with 5’ to 3’ direction away from the coverslip. Down-strands refer to strands designed with 5’ to 3’ direction towards the coverslip. Bold sequences represent the cognate binding sequences for the relevant repressor.

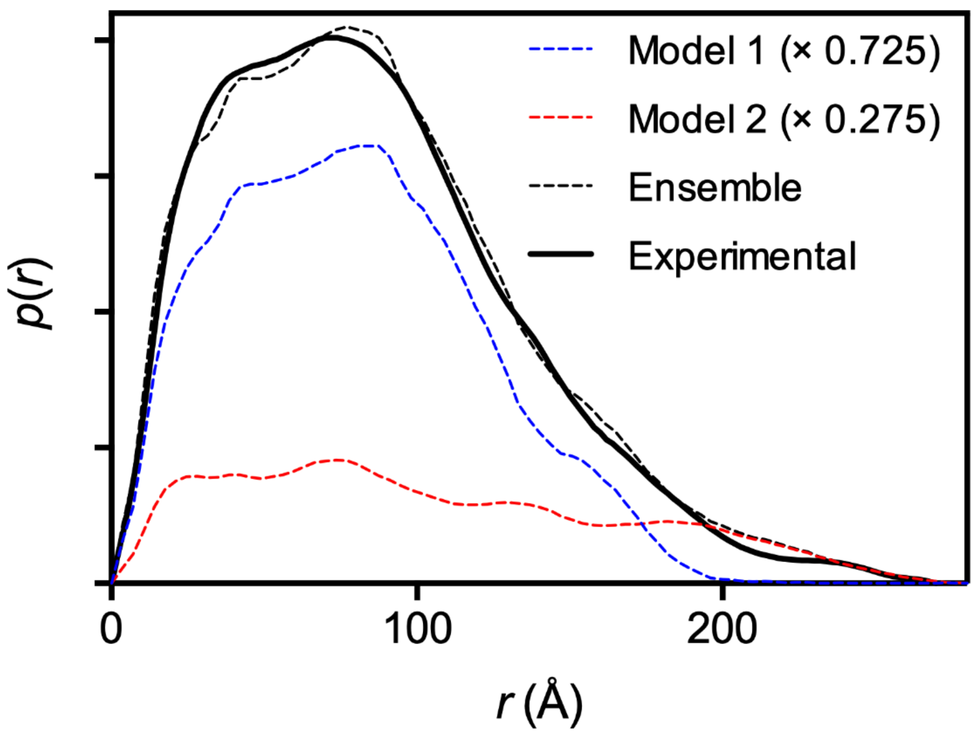

Supplementary Fig. 1. P(r) plot calculated from the SEC-SAXS data. The figure shows the pair-distance distribution function, $P(r)$, derived from the experimental data (solid black line). Overlaid are the $P(r)$ functions for the representative compact Model 1 (dotted blue line) and extended Model 2 (dotted red line) structures and their weighted sum representing the ensemble average (black dotted line).

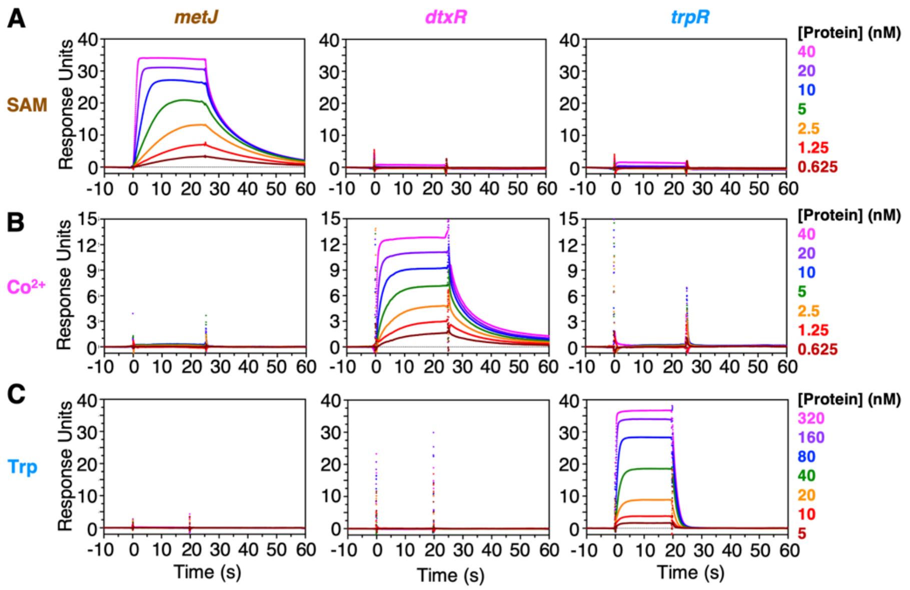

Supplementary Fig. 2. DNA binding by MetJ, DtxR and TrpR is orthogonal. MetJ, DtxR and TrpR were titrated onto each of their respective cognate sites via SPR in the presence of **(A)** SAM, **(B)** Co^{2+} and **(C)** Trp. The DNA sites used in these experiments are named above the figures, with sequences reported in Supplementary Table 5. The steady state responses were fit to the Hill equation, with the fitted binding parameters for each specific interaction reported in Extended Data Table 1.

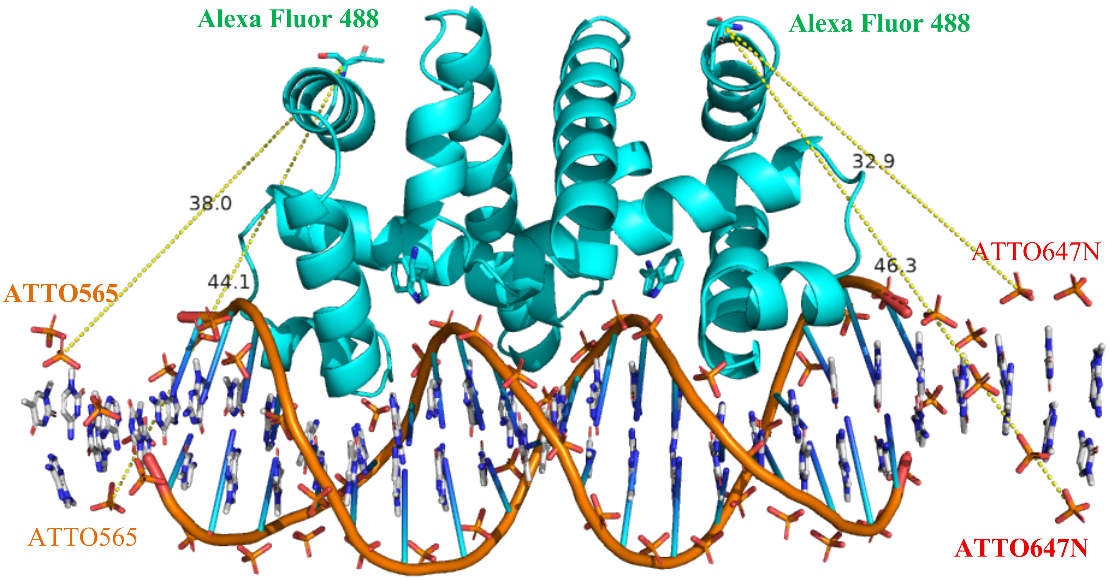

Supplementary Fig. 3. FRET donor–acceptor distances for TrpR. A model of a DNA sequence containing a *trpR* binding site and the relevant labelled flanking sequences per Supplementary Table 6. This DNA model is shown in stick representation. The DNA model was aligned to the DNA from a TrpR:DNA crystal structure (PDB: 1TRO, RMSD = 1.3 Å), with the crystal structure shown in cartoon representation. Distances were measured from the α-carbon of TrpR-S107, representing the donor dye position, to the phosphate of the labelled base (label bolded), representing the acceptor dye position. Distances are reported in Ångstroms. A second set of distances was measured between the donor site and the phosphate of the base complementary to the labelled base (label non-bolded), as TrpR can bind to either face of its palindromic binding site. The donor–acceptor distances are below the R_0 for both dyes (64.01 Å for ATTO565, 50.33 Å for ATTO647N, Supplementary Table 3).

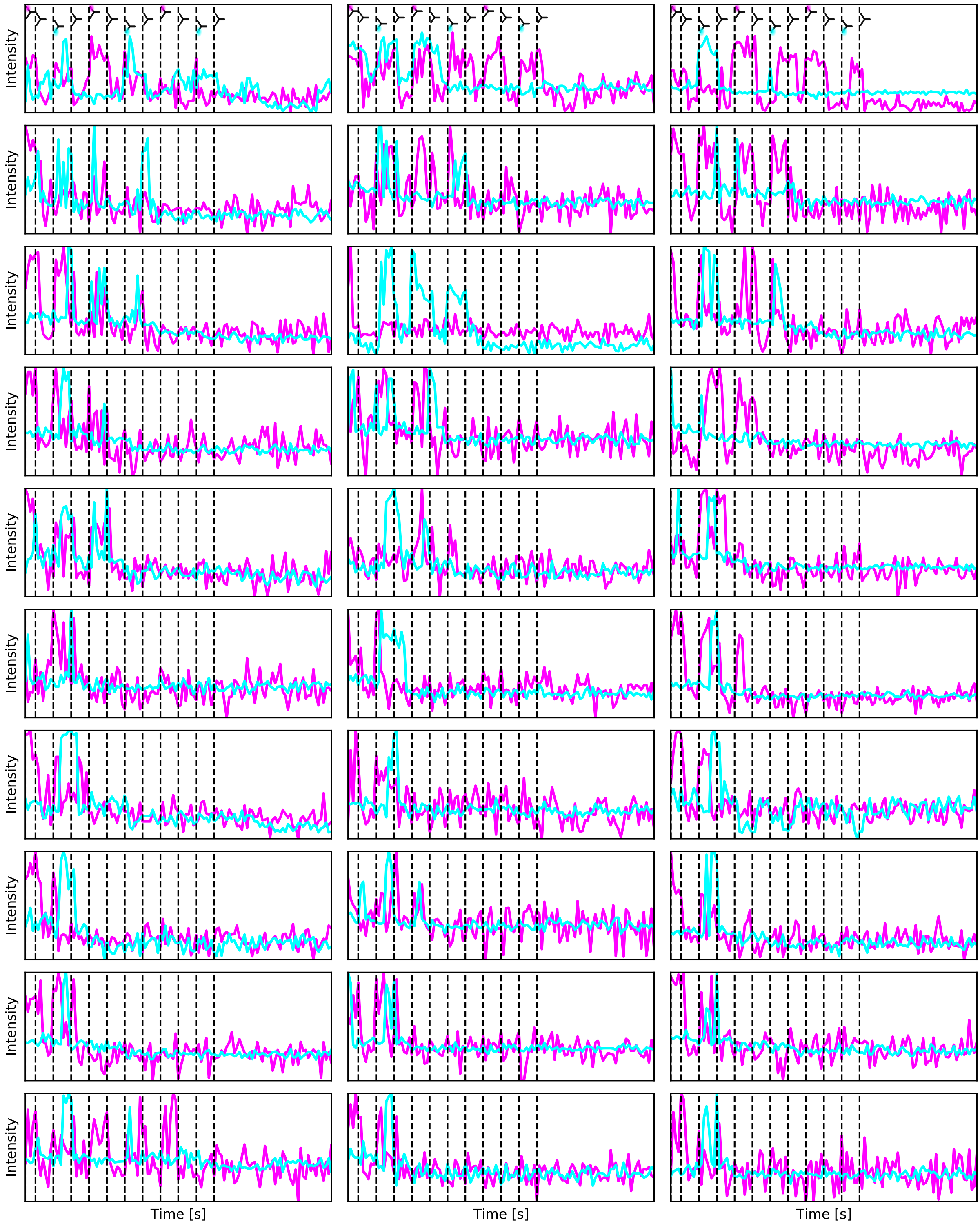

Supplementary Fig. 4. Normalized examples of single-molecule traces using two-colour imaging with emission split at 633 nm to simultaneously record ATTO565 emission (FRET emission from the bottom *trpR* site, shown in cyan) and ATTO647N emission (FRET emission from the top *trpR* site, shown in magenta). At the top of each column, a schematic of expected TW position and false colour representation of expected fluorescence signal given solution changes (dashed lines).

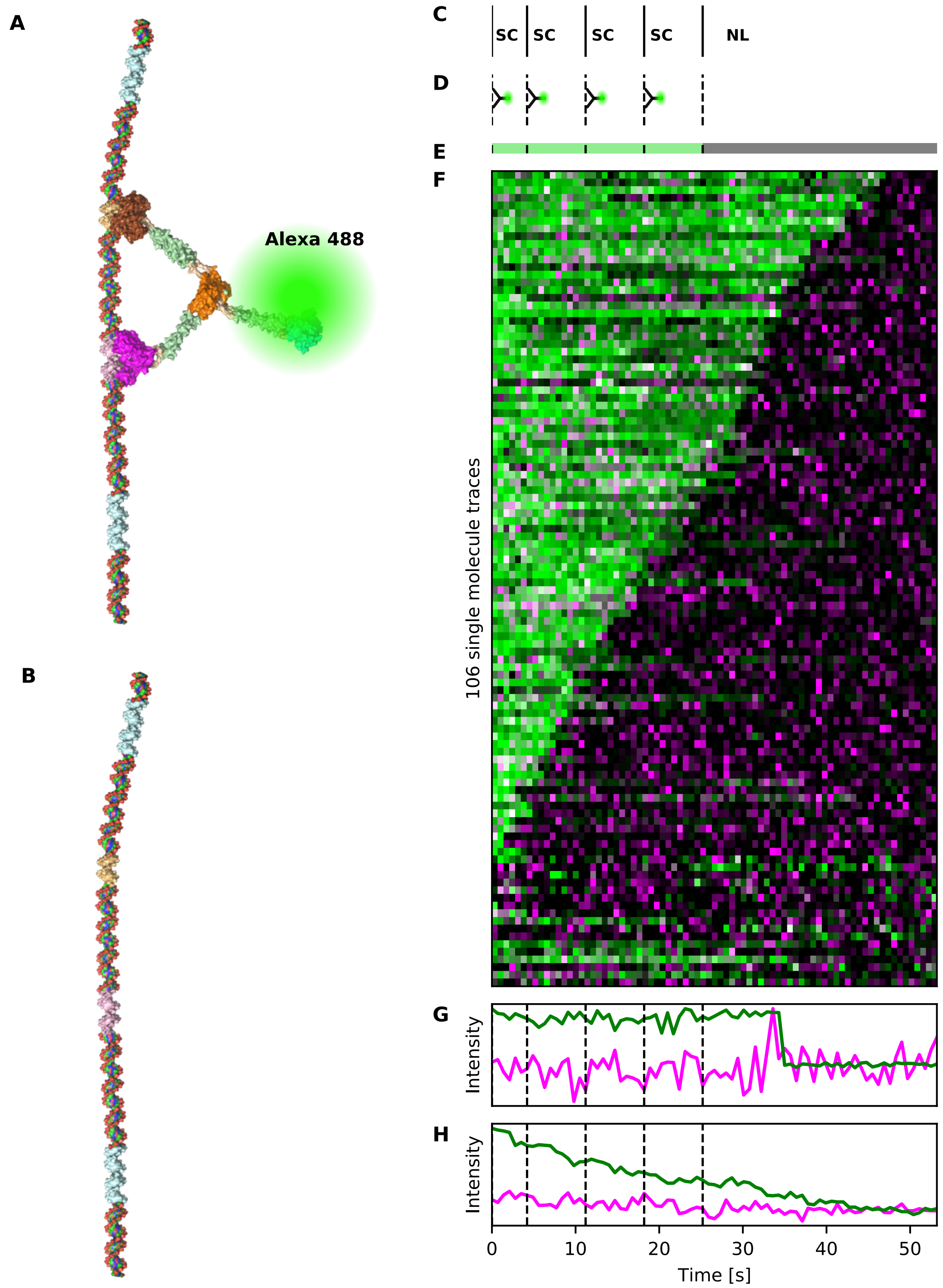

Supplementary Fig. 5. **TW remained static on DNA track when ligand combination (Co^{2+} + SAM) remained the same after microfluidic solution changes, as observed by single-molecule FRET.** (**A**) TW bound to the central *metJ-dtxR* sites emits green fluorescence while (**B**) no fluorescence is observed when TW detaches from the track. (**C**) sequence of fluidic changes between different fluids with the same concentrations of SAM and Co^{2+} (SC) before switching to no ligands (NL). (**D**) Cartoon showing expected TW position on the track. (**E**) Expected fluorescent signal as a function of fluidic changes. (**F**) Kymographs of all detected colocalizing single molecules sorted by time of last identified

fluorescence (106 single-molecule traces). **(G)** Normalized example single-molecule trace. **(H)** Sum of all single-molecule traces in the experiment.

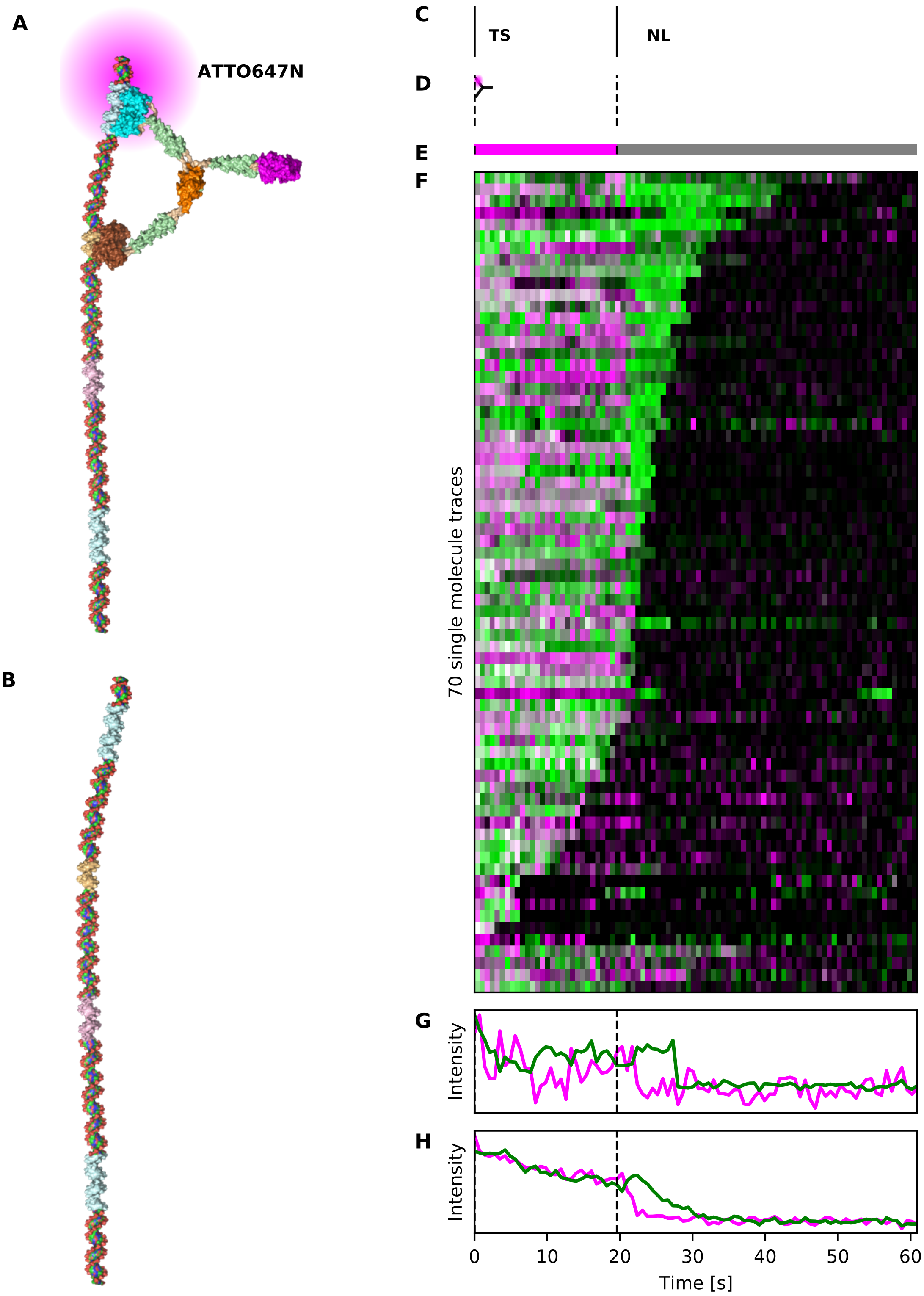

Supplementary Fig. 6. **TW rapidly detached from DNA track after removal of ligands (SAM + Trp), as observed by single-molecule FRET. (A)** TW bound to the top *trpR-metJ* sites emits magenta FRET signal while (**B**) no fluorescence is observed when TW detaches from the track. (**C**) Sequence of fluidic changes from Trp and SAM (TS) to no ligands (NL). (**D**) Cartoon showing expected TW position on the track. (**E**) Expected fluorescent signal as a function of fluidic changes. (**F**) Kymographs of all detected colocalizing single molecules sorted by time of last identified fluorescence (70 single-molecule traces). **(G)** Normalized example single-molecule trace. **(H)** Sum of all single-molecule traces in the experiment.

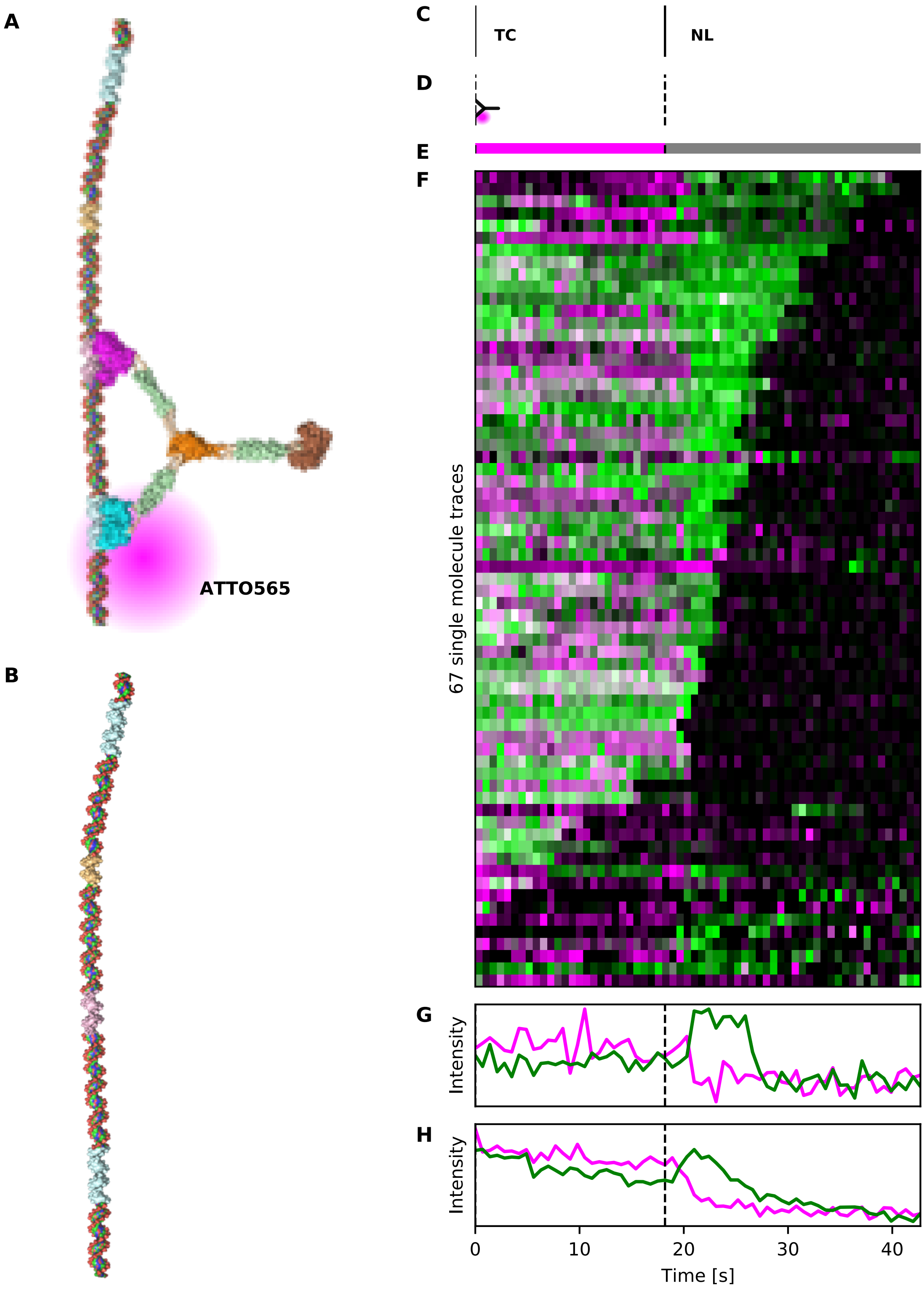

Supplementary Fig. 7. TW rapidly detached from DNA track after removal of ligands (Trp + Co^{2+}), as observed by single-molecule FRET. (A) TW bound to the bottom *dtxR-trpR* sites emits magenta FRET signal while (**B**) no fluorescence is observed when TW detaches from the track. (**C**) Sequence of fluidic changes from Trp and Co^{2+} (TC) to no ligands (NL). (**D**) Cartoon showing expected TW position on the track. (**E**) Expected fluorescent signal as a function of fluidic changes. (**F**) Kymographs of all detected colocalizing single molecules sorted by time of last identified fluorescence (67 single-

molecule traces). **(G)** Normalized example single-molecule trace. **(H)** Sum of all single-molecule traces in the experiment.

```
>TW1
GKDLVDTTEM YLRTIYELEE EGVTPLRARI AERLEQSGPT VSQTVARMER   50
DGLVVVASDR SLQMTPTGRT LATAVMRKHR LAERLLTDII GLDINKVHDE  100
ADRWEHVMSD EVERRLVKVL KGSSGGSGTL YEALKENEKL HKEIEQKDNE  150
IARLKKENKE LAEVAQGSSG GSGSSGDSAT HIKFSKRDED GKELAGATME  200
LRDSSGKTIS TWISDGQVKD FYLYPGKYTF VETAAPDGYE VATAITFTVN  250
EQGQVTVNGK ATKGDAHI
```

DtxR
Gemnin$_{\text{Coiled-Coil}}$
SpyCatcher
TrpR
SpyTag
MetJ

```
>TW2
GAQQSPYSAA MAEQRHQEWL RFVDLLKNAY QNDLHLPLLN LMLTPDEREA LGTRVRIVEE LLRGEMSQRE   70
LKNELGAGIA TITRGSNSLK AAPVELRQWL EEVLLKSDGS SGGSGTLYEA LKENEKLHKE IEQKDNEIAR 140
LKKENKELAE VAQGSSGGSG SSGAHIVMVD AYKPTKGSSG GSGSSGTLYE ALKENEKLHK EIEQKDNEIA 210
RLKKENKELA EVAQGSSGGS GGAEWSGEYI SPYAEHGKKS EQVKKITVSI PLKVLKILTD ERTRRKVNNL 280
RHATNSELLC EAFLHAFTGQ PLPDDADLRK ERSDEIPEAA KEIMREMGIN PETWEY
```

Supplementary Fig. 8. Protein sequences of the TW constructs. Protein domains are colored per the top right inset. Glycine-serine rich linkers are colored grey. TW is formed via the formation of an isopeptide bond between the side chains of K186 in the SpyCatcher domain of TW1 and D170 in the SpyTag part of TW2. The S107C mutation was introduced into the TrpR domain of TW2 to enable labelling of the protein with Alexa Fluor 488 via maleimide chemistry.

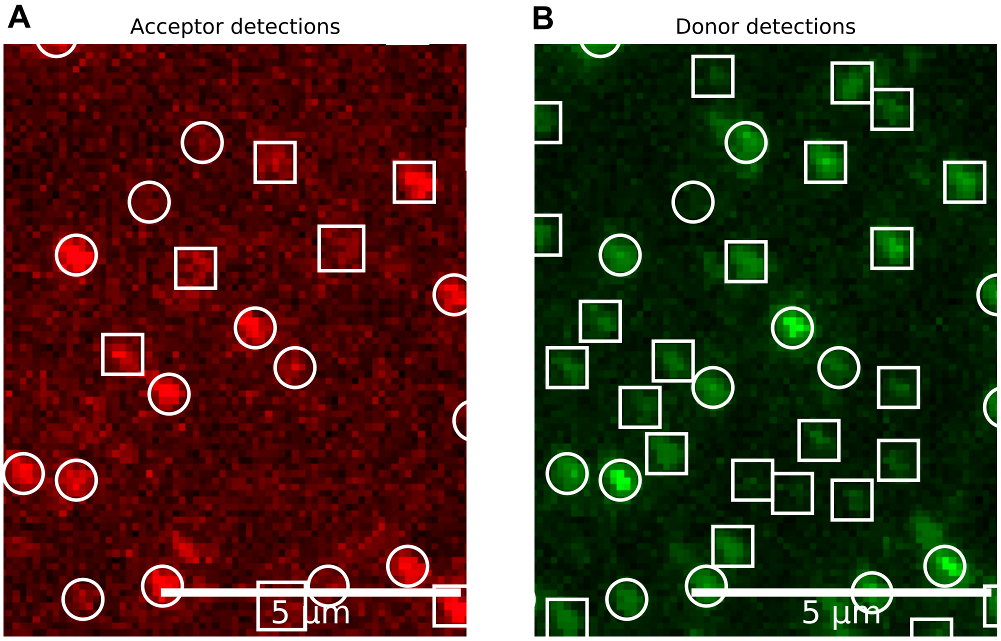

Supplementary Fig. 9. Example of detected bright spots. A small field of view from a normalized maximum intensity projection of the data from **(A)** the FRET acceptor channel and **(B)** the donor channel from Experiment 1. All bright spots detected by the algorithm are outlined with white squares or circles. Squares denote those detected spots that were identified as not colocalizing in Donor and FRET channels. Circles denote those detected spots that were identified as colocalizing in Donor and FRET channels.

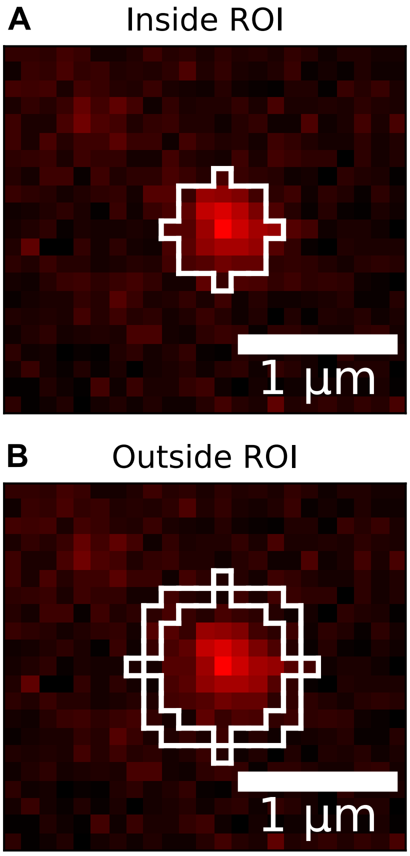

Supplementary Fig. 10. Regions of interest created for single-molecule analysis. (A) shows the pixels used to determine intensity of a spot, and **(B)** shows the surrounding pixels whose intensity is used for background removal. The final background subtracted intensity of the molecular fluorescence signal in each frame is calculated by averaging the intensity of all pixels in the region of interest in **(A)** and subtracting the median in **(B)**.

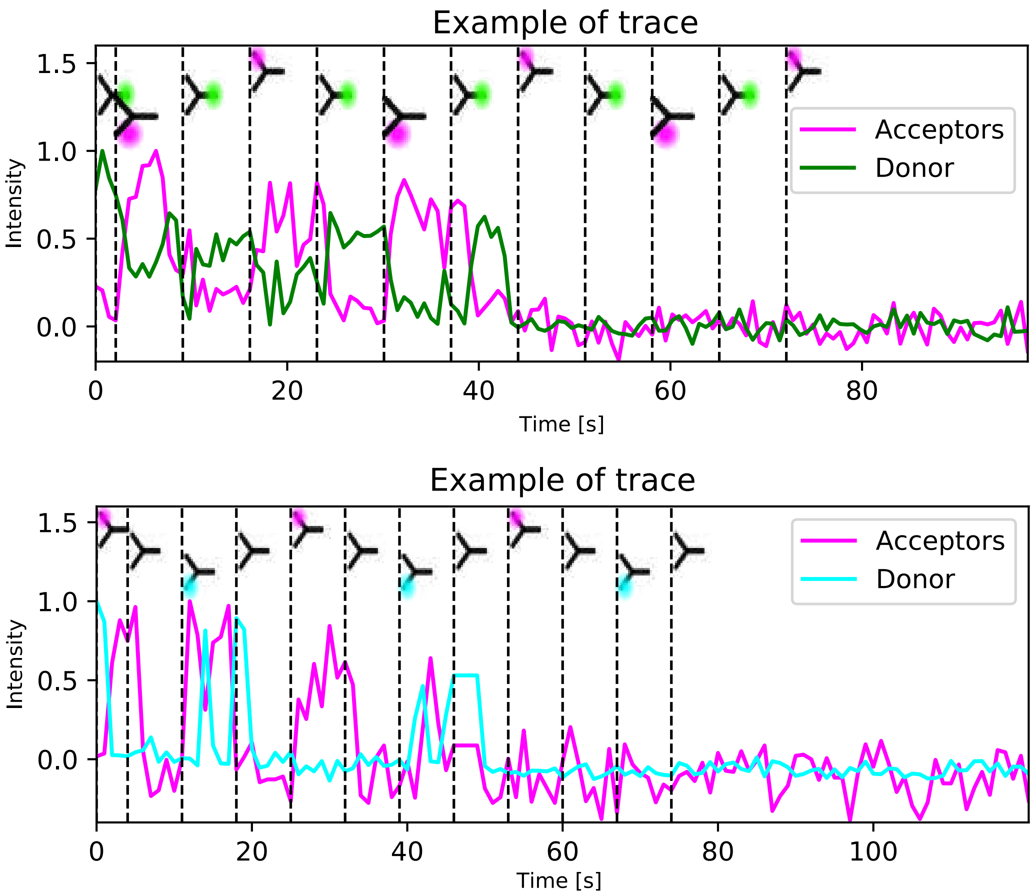

Supplementary Fig. 11. The resulting traces after background subtraction and normalization. Top: Experiment 1. Magenta is the FRET acceptor signal, green the donor. Black dashed lines indicate times of buffer changes. Bottom: Experiment 2. Magenta is the FRET acceptor signal from the red acceptor (top-*trpR* site) and cyan is the FRET acceptor signal from the yellow acceptor (bottom-*trpR* site). Black dashed lines indicate buffer switches.

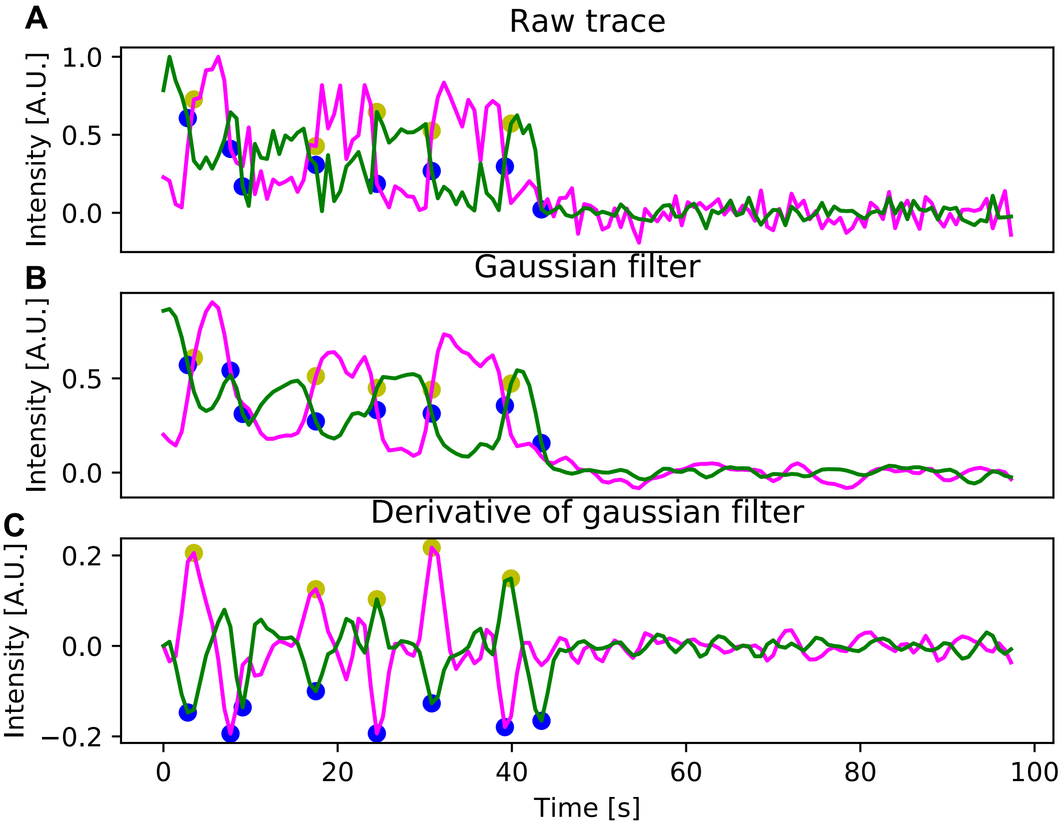

Supplementary Fig. 12. Outline of the edge detection algorithm. **(A)** shows the raw traces, **(B)** the raw traces after Gaussian filtering with standard deviation 1.2 seconds and **(C)** shows the derivative of **(B)**. The threshold for peak detection is indicated by a yellow dashed line and the threshold for valley detection is indicated by a blue dashed line. In all panels, the magenta is the FRET signal and the green is the donor signa. The detected rising edges are marked with a yellow dot and detected falling edges with a blue dot.

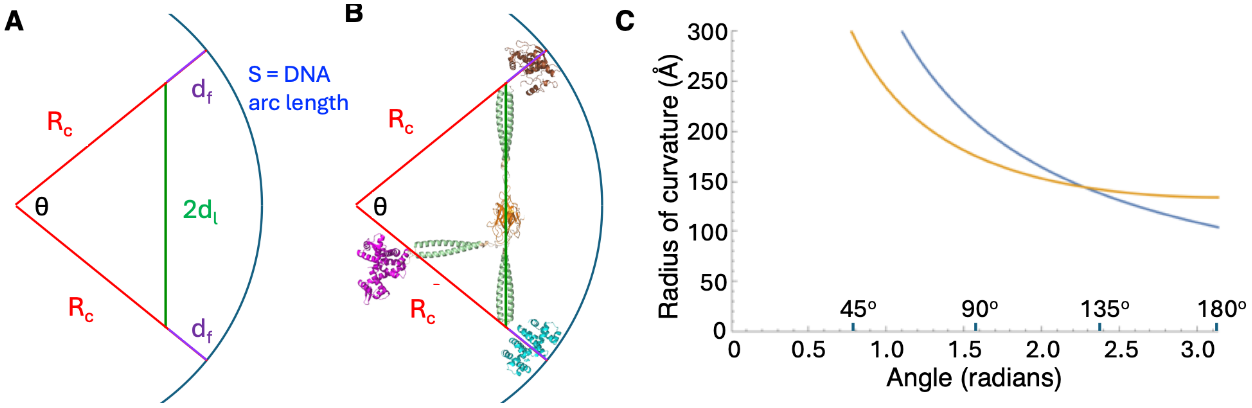

Supplementary Fig. 13. Model for TW overstepping due to DNA flexibility. (A) The geometry of DNA bending into a uniform circular arc. TW is represented by two stretched legs, total length $2d_l$ that connect to DNA via two repressor feet, each of length, d_f, that bind to DNA in a perpendicular fashion, each tracing a radius of curvature. **(B)** An overlay of a model of fully stretched TW on the geometric figure from (**A**). (**C**) Plot of the equation for the radius of curvature describing the DNA geometry (blue) and the TW geometry (orange) as a function of angle. The intersection of these two curves marks a simultaneous solution to the two equations.

Supplementary

Supplementary Discussion A

Estimation of TW dissociation rates, TW performance and non-specific binding

We have used a DNA displacement assay to study the TW:DNA dissociation kinetics in various ligand conditions. We use a large excess of free DNA in solution during the dissociation phase of the SPR experiment to prevent TW from rebinding to surface-bound DNA. Without free DNA in solution, the apparent half-lives of TW:DNA interactions are increased as TW fails to escape the surface of the SPR chip. By titrating the amount of free DNA, we show that 1 μM DNA is sufficient for reducing TW rebinding in the presence of each of the individual ligands (Extended Data Fig. 2C).

The individual TW feet dissociate from DNA on the timescale of seconds, thus, in the presence of two ligands, TW will not have both feet simultaneously bound to the DNA for the entire duration of its time bound to the track. Individual feet will transiently detach and reattach, until both feet happen to detach simultaneously, allowing TW to dissociate from the track. The longer TW remains in a single foot-bound state, the more vulnerable it is to dissociating from the track. In our displacement assay we provide an opportunity for a transiently unbound TW foot to catch a free DNA from the solution, resulting in a TW bound to the track through a single foot, increasing the likelihood that TW dissociates from the track. Therefore, our apparent half-lives for TW:DNA complexes in the presence of two ligands represent the lower-bound of time TW can remain bound to a track.

Regarding non-specific binding, a 2:1 TW:*dtxR-metJ* was found to form in the presence of the non-cognate ligand Trp via mass photometry (Extended Data Fig. 3C; Supplementary Table 1). This occurred at high TW concentrations (25 nM), with no evidence of a 1:1 TW:*dtxR-metJ* DNA complex in the presence of Trp, consistent with the cooperative binding seen in the SPR data (Hill coefficient of 1.7, Extended Data Table 1). Together, SPR and mass photometry results show that TW has a strong preference for forming specific, 1:1 complexes with DNA, and does not crosslink separate DNA molecules.

Supplementary Discussion B

Single-molecule FRET: Control Experiments

The effect of the fluid exchange in the absence of ligand changes was tested by performing the same biochemical preparation, imaging and mechanical flow switching as in the stepping experiments, but instead with Co^{2+}+ SAM buffer in each of the three microfluidic reservoirs. Supplementary Figure 5 shows traces obtained using a single-molecule FRET analysis. In this experiment the TW is never expected to bind to a site labelled with a FRET acceptor, so the analyses for colocalization was relaxed by removing the requirement for colocalization with FRET intensity. Instead, relying only on colocalization with detections in the acceptor excitation channel. Keeping the FRET requirement removes all molecules from the analysis as expected. Otherwise, analysis was performed as in the above Methods section. Solutions were changed between identical Co^{2+}+ SAM buffer 3 times and then changed to a buffer containing no ligands at all as indicated by Supplementary Figure 5C. Supplementary Figure 5G shows an example of these traces where TW dissociates from the DNA after the removal of ligands. All traces are shown as overlayed kymographs in Supplementary Figure 5F where green is TW donor signal and magenta is *trpR* site FRET. The kymograph shows no FRET nor any reaction to the changes in flow at all. At the point at which all ligands are removed (Supplementary Fig. 5C) a slower than anticipated rate of signal loss is observed (Supplementary Fig. 5F and G). This is due to the relaxed colocalization criteria allowing non-specifically bound nearby TW and DNA and potentially non-functional TW:DNA complexes to be included in the analysis. This is particular to this control experiment where we do not have a FRET signal as an analysis criterion.

Similar detachment experiments were performed with Trp + Co^{2+} (Supplementary Fig. 6) and SAM + Trp, (Supplementary Fig. 7), with a single solution change to the ligand-free solution. When changing to a ligand-free solution, the rate of TW detachment increases sharply for both of these ligand-pair conditions.

Supplementary Discussion C

Simulation of TW Stepping

The single-molecule FRET data in Figs 4 and 5 clearly demonstrate that TW can step bi-directionally on a short track consisting of four binding sites, but does not allow us to directly and experimentally evaluate performance parameters such as processivity and average speed on a long track. However, experimentally measured binding and stepping characteristics allow us to predict this performance using a model.

To analyze potential stepping behavior of TW along an extended track we devised a coarse-grained model based on Master equations governing the binding and unbinding kinetics of the three TW feet and the track. The track is represented by the periodic, unlimited sequence of binding sites …-*dtxR-metJ-trpR-dtxR-metJ-trpR-dtxR-metJ*-… (Extended Data Fig. 5), mimicking the experimental setup used in Figs 4 and 5. The kinetics of the three individual feet are described by three independent rate equations. The rate equation for each foot describes the transitions between an unbound state and bound states, in which the foot is bound specifically to its matching binding site on the track, e.g., the DtxR foot can only bind to *dtxR* etc., while non-specific binding is neglected, supported by the SPR results in Fig. 3A. Since these equations govern transitions between states (not concentrations), the corresponding rates have units [1/time] and are the inverse of the average waiting time between transitions. The values of these rates depend on the ligands in solution (see Extended Data Table 2), which are cycled periodically through the combinations Trp + Co^{2+}, Co^{2+} + SAM, SAM + Trp, with a period of 21 seconds (i.e. the solution is switched every 7 seconds). Since there are always two ligands present at any given time, the rate equations for the individual feet become effectively coupled.

As an example, the set of possible states and the considered transitions between them are shown in Extended Data Fig. 5 for the ligand combination Co^{2+} + SAM. In particular, Extended Data Fig. 5 illustrates that we set up the rate model for each foot to distinguish between two distinct bound states, representing binding of a foot to its intended site (adjacent to the other bound foot) and unintended binding to the non-adjacent binding site (overstepping). For the other ligand combinations, Trp + Co^{2+} and SAM + Trp, analogous state combinations and transitions are realized by the rate equations.

The dynamics of each foot is thus driven by four rates for the transitions between its unbound state and two distinct bound states, which all depend on the foot-specific ligand being present in the solution or not. Fixing the explicit values for these rates in the model is guided by the experimental findings and is described below.

The rate processes for the three TW feet, their dependence on the ligand combination present in solution, and the 21-second cycle of the solution through three ligand combinations were implemented in a custom-built C++ code. A simulation run started with two feet bound on the track in the presence of the corresponding ligand combination, and ran until the TW detached from the track, *i.e.*, until none of the feet was in a bound state. The sequence of binding and unbinding events recorded during such a run for all three feet was translated into the corresponding stepping motion of the TW along the track. This resulted in a trajectory of the TW showing the position of its center (in units of track periods) as a function of time (in seconds). Examples of such trajectories are shown in Extended Data Fig. 6.

Repeating simulations for individual trajectories many times gives access to the distribution of observables with which the expected stepping performance of the TW along an extended

track can be characterized. We focus on the stepping distance as a function of time (giving the stepping velocity, Extended Data Fig. 6A), the variance of the trajectories as a function of time (giving the effective diffusion coefficient, Extended Data Fig. 6B), and the distance covered before the TW detaches from the track (Extended Data Fig. 6E & F).

The specific values for the various rates of the individual TW feet in our model were chosen as follows. We first focus on the rates describing (un-)binding between the TW repressor feet and the intended binding site (adjacent to the other bound foot): The off-rates for each repressor in the presence and absence of its ligand were adapted from the SPR experiments, see Extended Data Table 2, *i.e.*, the off-rates are characteristic for the type of repressor (TrpR, DtxR, MetJ). To choose the on-rates, we assumed that (re-)binding to the track is predominantly determined by the geometrical arrangement of the track and the binding sites, and thus can be assumed to be independent of the repressor type. For this reason, we took on-rates to be identical for all feet. Moreover, neglecting non-specific binding, the on-rates are all zero in the absence of the foot's ligand (Extended Data Table 2). In the presence of ligands, we adjusted the value of the on-rates such that the three *double-ligand* off-rates measured in the SPR dissociation experiments for the ligand combinations Trp + Co^{2+}, SAM + Co^{2+}, and SAM + Trp (Extended Data Table 2) were best reproduced. To do this, we ran 10^6 simulations for each pair of feet and corresponding ligands, with the assumed value of the on-rates, until neither of the feet was bound . From the exponential distribution of the so obtained "survival times" we extract the effective dissociation rate; the results are listed in Extended Data Table 2.

For unintended binding to the non-adjacent site (overstepping), we assumed the off-rates to be identical to the ones for unbinding from the intended binding site, because the off-rates are characteristic for the type of repressor, no matter where on the track it is bound. In the spirit of our assumption that the binding process is predominantly geometry-determined, the on-rates are again all the same for the different feet, but in general different from the on-rates to the intended binding site (different geometry). We encoded the difference in a factor a that multiplies the on-rates for intended binding:

$$\text{(on-rates for overstepping)} = \alpha * \text{(on-rates for intended binding)}. \qquad 8$$

For $\alpha = 0$, there is no overstepping, $0 < \alpha < 1$ implies that overstepping is less likely than intended binding, while $\alpha > 1$ indicates that overstepping is more likely; for $\alpha = 1$ intended binding and overstepping are equally likely. The relative weight of overstepping is calculated by comparing its rate to the total rate of binding, $\alpha/(1+\alpha)$. Hence, for $\alpha = 0.5$ we find 1/3 = 33%, reproducing the experimentally observed frequency of overstepping of about 35%.

Supplementary Discussion D

Fall-off versus Photobleaching in Kymographs

Is it possible to disentangle TW fall-off and fluorophore bleaching in the kymographs (Figs. 4G and 5G)? To address this question, we used our coarse-grained model (see Supplementary Discussion C) to reproduce Experiment 1 (Fig. 4) and Experiment 2 (Fig. 5), in which TW walks back-and-forth along a four-site DNA track. We simulated as many independent realizations for each experiment as there are traces in the corresponding kymographs (322 realizations for Experiment 1, see Fig. 4G; 236 realizations for Experiment 2, see Fig. 5G), and recorded the "fall-off times" when the TW molecule dissociates from the track.

To mimic photobleaching we assumed that bleaching occurs with a constant rate. This implies that the times at which the fluorophore bleaches are exponentially distributed with a "mean bleaching time" which is given by 1/(bleaching rate). For each simulated TW trace, we determined its bleaching time by drawing a random number from an exponential distribution with a given mean bleaching time. We then cut off the trace at the bleaching time if bleaching occurs before fall-off, otherwise we kept the full length of the TW trace. Finally, we sorted the traces from shortest to longest and plotted the bleached fall-off times as a "fall-off/bleaching front" as per Figs. 4G and 5G.

In experiment 2 (Fig. 5G), there are dark intervals when the TW is at the *metJ-dtxR* position in the middle of the track; in the simulations, these "dark states" have been taken into account by cutting off times to the last time point with a fluorescence signal before fall-off.

The resulting plots should then approximate the edge in the kymographs at which the signal becomes dark. Extended Data Fig. 7 shows simulations of the kymograph for Experiment 1 (Extended Data Fig. 7 A–C) and for Experiment 2 (Extended Data Fig. 7D–F). For Experiment 1, a typical kymograph with a bleaching time of 200 seconds is overlaid on the experimental kymograph in Extended Data Fig. 7A (white curve); Extended Data Fig. 7B shows 10 independent simulations using identical parameters to Extended Data Fig. 7A, which all share the same typical features. Finally, Extended Data Fig. 7C shows the effect of varying the bleaching time (the different curves are labelled with their bleaching times), and we included the curve without bleaching (in orange). For Experiment 2, the corresponding plots are shown in Extended Data Fig. 7D-F.

The simulated kymographs (Extended Data Fig. 7A & D) reproduce the features of the experimental kymographs (Figs 4G and 5G). From visual inspection, a bleaching time of 200 seconds best approximates the experiment.

Supplementary Discussion E

Can DNA bend sufficiently to facilitate overstepping?
The inherent flexibility of the DNA track may be responsible for the observed overstepping of TW. To test this possibility, we used a simple physical model to determine the energetic cost of bending the DNA sufficiently to allow overstepping, *i.e.*, for two repressor feet to bind sites separated by an intervening empty site.

If DNA is uniformly flexible, then it can follow a circular arc described by a radius of curvature, R_c, an arc length, s, and the angle subtended by the arc, θ, where:

$$R_c = \frac{s}{\theta}. \qquad (5)$$

The largest possible reach of two TW feet occurs when the two legs (each of length d_l) are straight and each repressor foot (additional length d_f) binds to DNA perpendicularly (thus aligning the foot's axis with the local radius of curvature) (Supplementary Figs 13A & B; similar to Model 2 in Fig. 2C). For an angle θ subtended by the feet, this configuration of TW can be described by:

$$sin\left(\frac{\theta}{2}\right) = \frac{d_l}{R_c - d_f}. \qquad (6)$$

From our model of TW, we approximate: $d_l = 100$ Å and $d_f = 35$ Å. From our model of the DNA track, the arc length between two non-consecutive repressor binding sites is approximately $s = 330$ Å. Using these parameters, we can plot equations 5 and 6 for radius of curvature, R_c, as a function of the angle θ subtended – the equation 5 describing the DNA arc and equation 6 describing TW. The intersection of these two curves represents the solution of these two geometric equations (Supplementary Fig. 13C), where $R_c \cong 145$ Å and $\theta \cong 130°$.

By modelling DNA as a rigid rod of length s (reference [6]), we can estimate the energy, ΔU, required to bend DNA sufficiently to satisfy this geometric solution:

$$\Delta U = \frac{1}{2}\frac{BLs}{R_c^2}, \qquad (7)$$

where B is the bending stiffness, $B = 50nm.k_BT$ [6]. This gives $\Delta U = 3.9k_BT$, where k_B is the Boltzmann constant. Thus, it would not require a large thermal fluctuation to achieve the required bending of the DNA that would facilitate overstepping (along with flexibility in the TW linker regions). This bending could be reduced by supporting the DNA track in a straight configuration.

References

1 Vandenberk, N., Barth, A., Borrenberghs, D., Hofkens, J. & Hendrix, J. Evaluation of Blue and Far-Red Dye Pairs in Single-Molecule Forster Resonance Energy Transfer Experiments. *J Phys Chem B* **122**, 4249-4266 (2018). https://doi.org:10.1021/acs.jpcb.8b00108

2 Hellenkamp, B. *et al.* Precision and accuracy of single-molecule FRET measurements-a multi-laboratory benchmark study. *Nat Methods* **15**, 669-676 (2018). https://doi.org:10.1038/s41592-018-0085-0

3 Sharma, R., Patelli, A. S., De Bruin, L. & Maddocks, J. H. cgNA+web : A Visual Interface to the cgNA+ Sequence-dependent Statistical Mechanics Model of Double-stranded Nucleic Acids. *J Mol Biol* **435**, 167978 (2023). https://doi.org:10.1016/j.jmb.2023.167978

4 Petoukhov, M. V. *et al.* New developments in the ATSAS program package for small-angle scattering data analysis. *J Appl Crystallogr* **45**, 342-350 (2012). https://doi.org:10.1107/s0021889812007662

5 Schneidman-Duhovny, D., Hammel, M., Tainer, J. A. & Sali, A. FoXS, FoXSDock and MultiFoXS: Single-state and multi-state structural modeling of proteins and their complexes based on SAXS profiles. *Nucleic Acids Res* **44**, W424-429 (2016). https://doi.org:10.1093/nar/gkw389

6 Marko, J. F. & Cocco, S. The micromechanics of DNA. *Physics World* **16**, 37 (2003). https://doi.org:10.1088/2058-7058/16/3/40
