# Supplementary figures and images for: Clocked stepping of an artificial protein walker along a DNA track
Source: Nat Nanotechnol. 2026 Jul 6;21(7):987–95. doi: 10.1038/s41565-026-02211-3 (PMC13379313; doi:10.1038/s41565-026-02211-3)

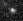

Supplement: Supplementary file 3 — Source data for Supplementary single-molecule data—Supplementary Figs. 4–7 and 9–12. [file 41565_2026_2211_MOESM3_ESM.zip › Nilsson_Supplementary_Data_2/Fig_S10.tiff]

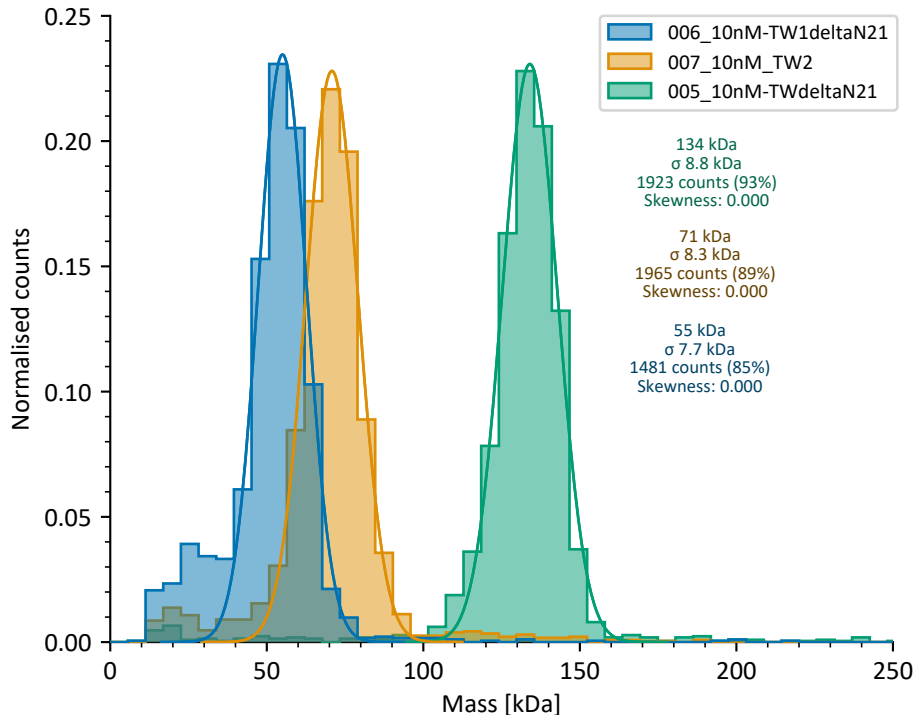

Supplement: Supplementary file 4 — Excel spreadsheet of molecular masses, SAXS and Guinier plot; unprocessed SDS–PAGE image, mass photometry data output and AFM image. [file 41565_2026_2211_MOESM4_ESM.zip › Figure2_SourceData/Figure2B_SourceData.pdf]

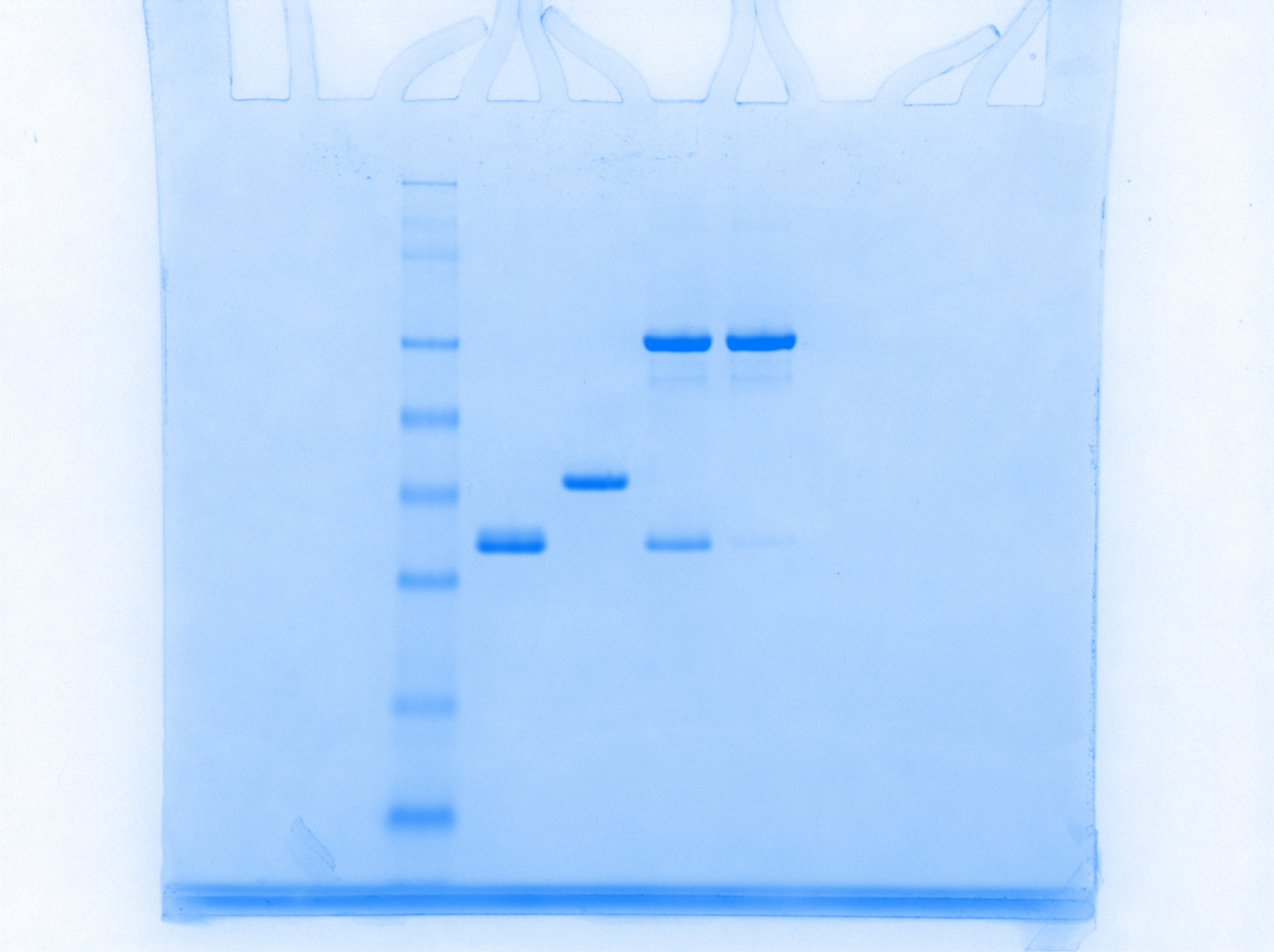

Supplement: Supplementary file 4 — Excel spreadsheet of molecular masses, SAXS and Guinier plot; unprocessed SDS–PAGE image, mass photometry data output and AFM image. [file 41565_2026_2211_MOESM4_ESM.zip › Figure2_SourceData/Figure2A_SourceData.tif]

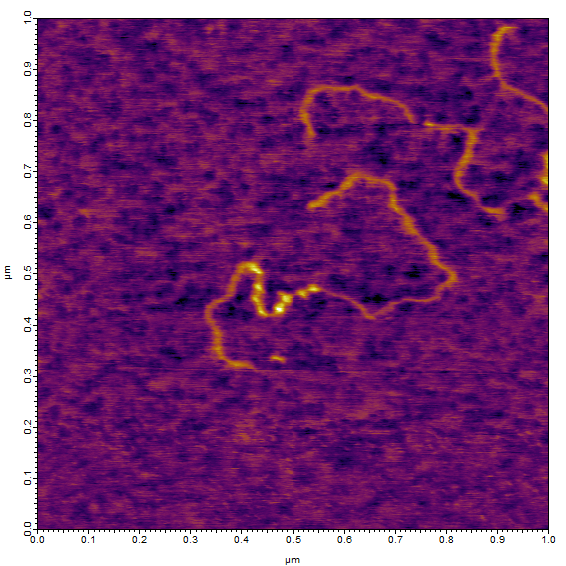

Supplement: Supplementary file 4 — Excel spreadsheet of molecular masses, SAXS and Guinier plot; unprocessed SDS–PAGE image, mass photometry data output and AFM image. [file 41565_2026_2211_MOESM4_ESM.zip › Figure2_SourceData/Figure2D_SourceData.tif]

# SAMTrp

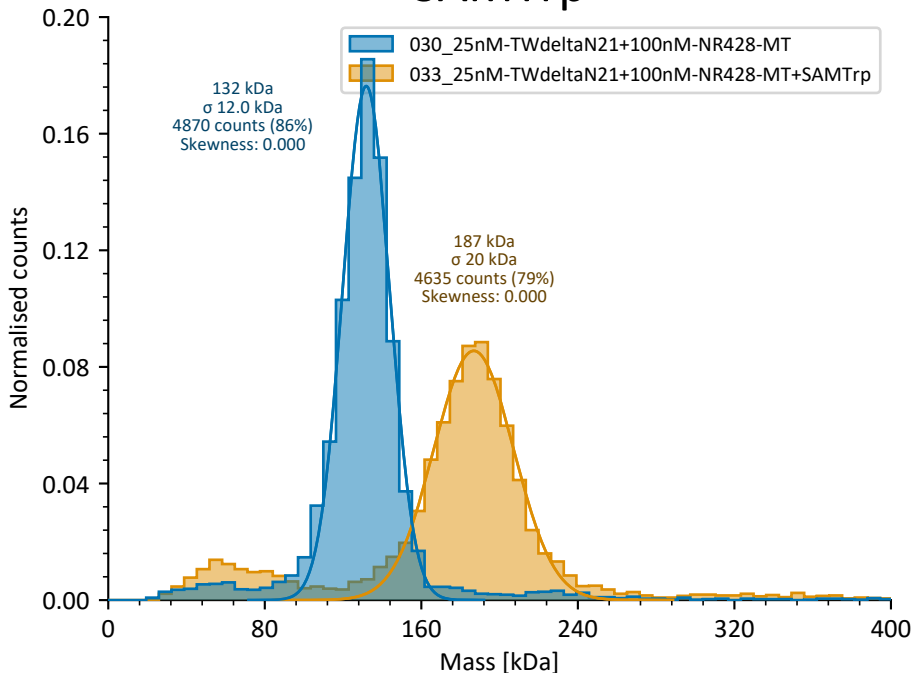

Supplement: Supplementary file 5 — Excel spreadsheet of SPR and mass photometry data; mass photometry data output. [file 41565_2026_2211_MOESM5_ESM.zip › Figure3_SourceData/Figure3C_SourceData.pdf]

# SAM

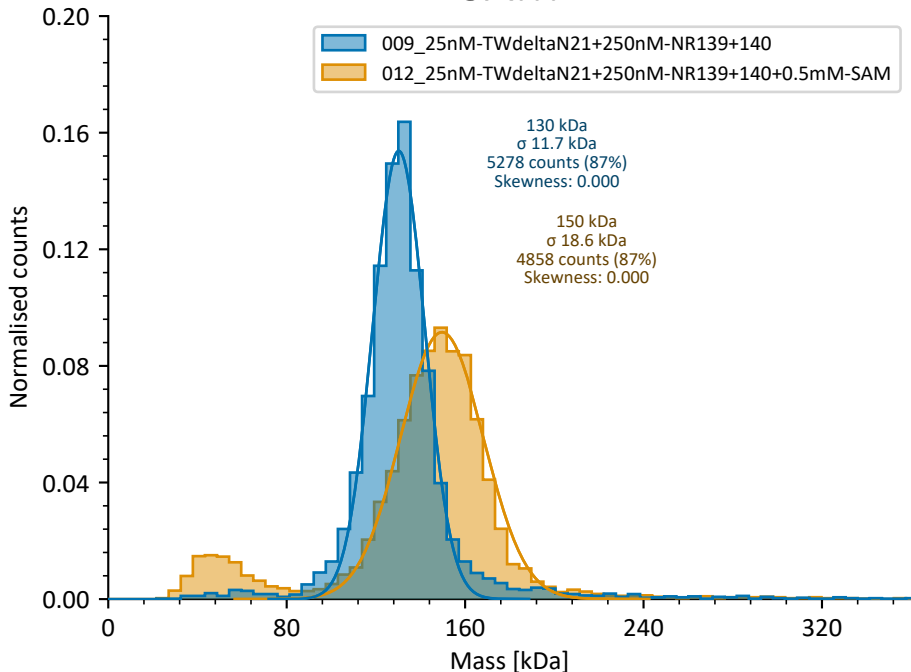

# CoCl<sub>2</sub>

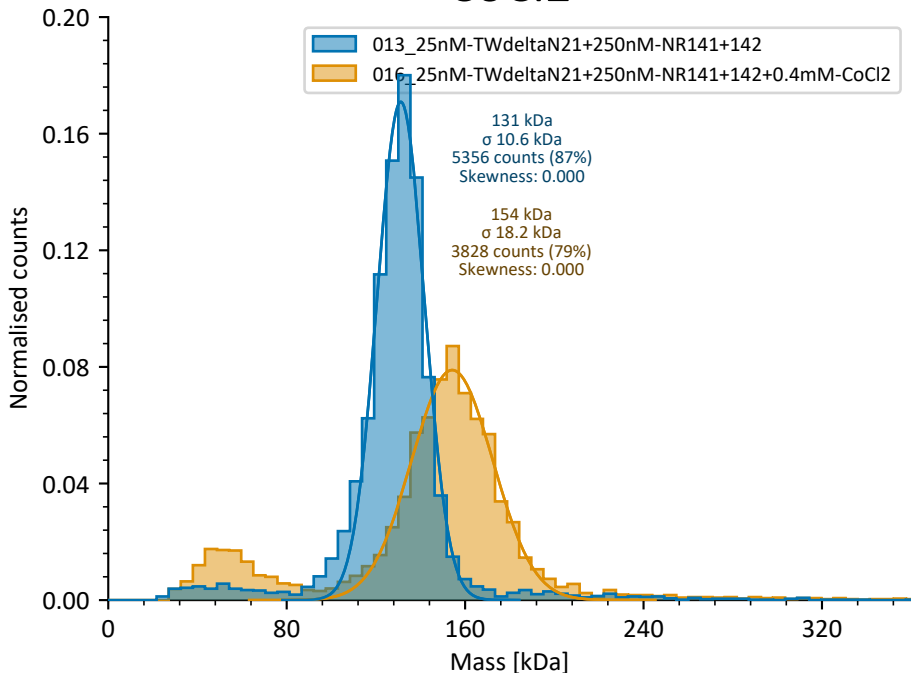

# Trp

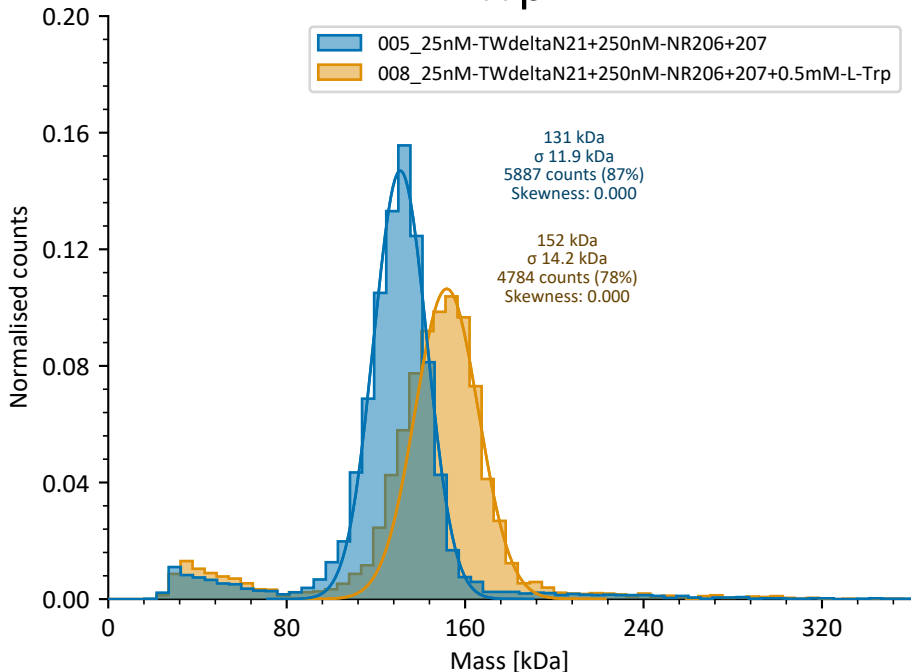

Supplement: Supplementary file 12 — Mass photometry output data. [file 41565_2026_2211_MOESM12_ESM.zip › Extended_Data_Figure_3_Source_Data/ExtendedFig3A_SourceData.pdf]

# CoTrp

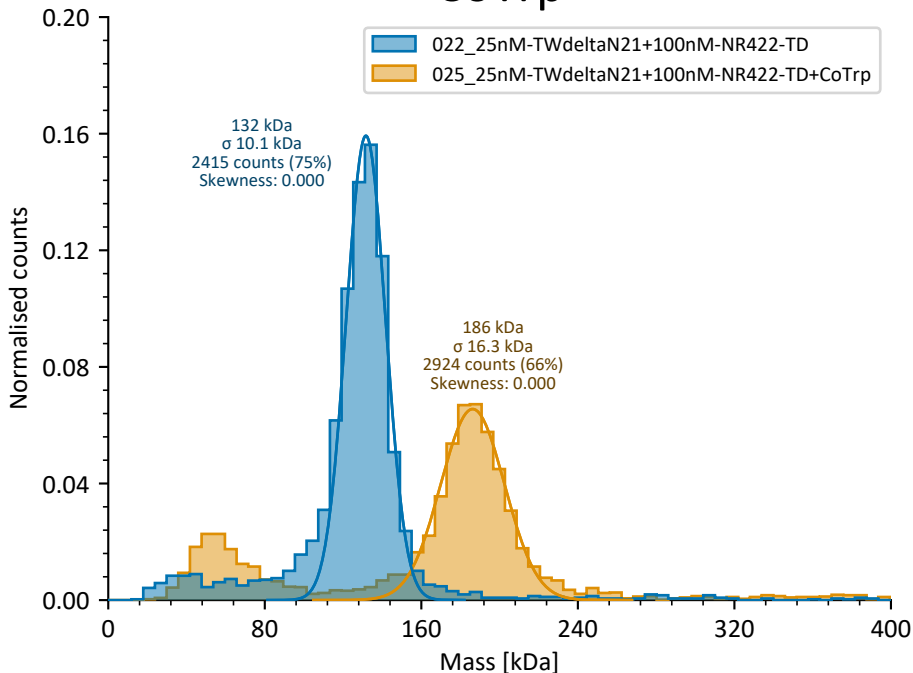

# SAMCo

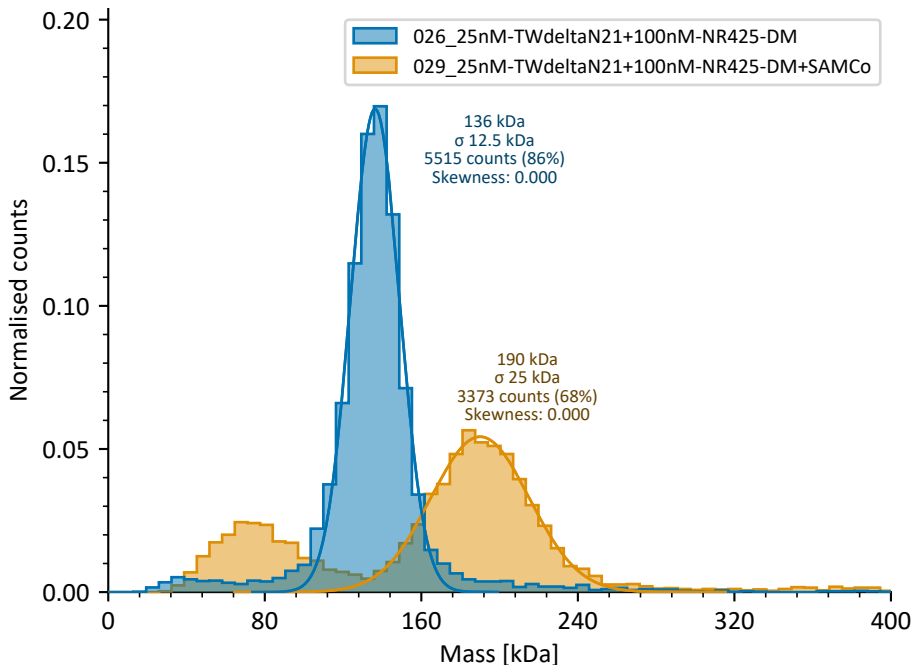

# SAMTrp

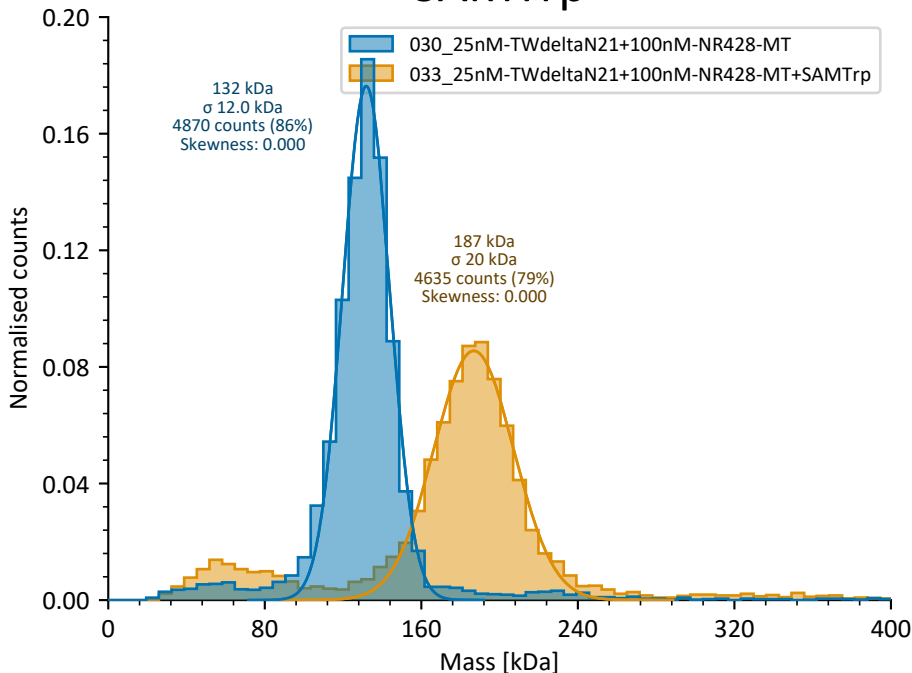

Supplement: Supplementary file 12 — Mass photometry output data. [file 41565_2026_2211_MOESM12_ESM.zip › Extended_Data_Figure_3_Source_Data/ExtendedFig3B_SourceData.pdf]

# NR422+Trp

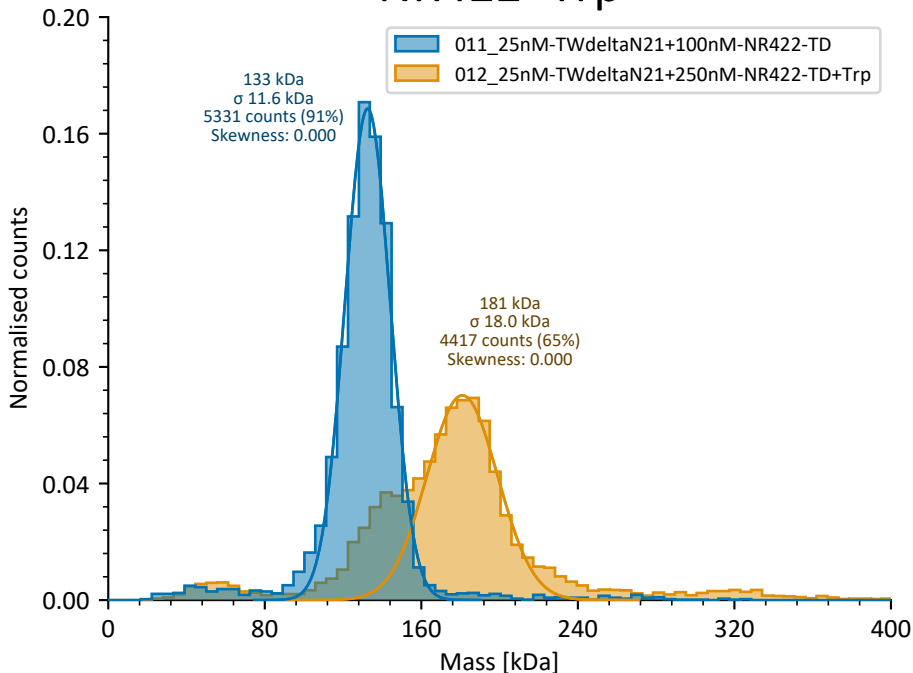

# NR425-DM

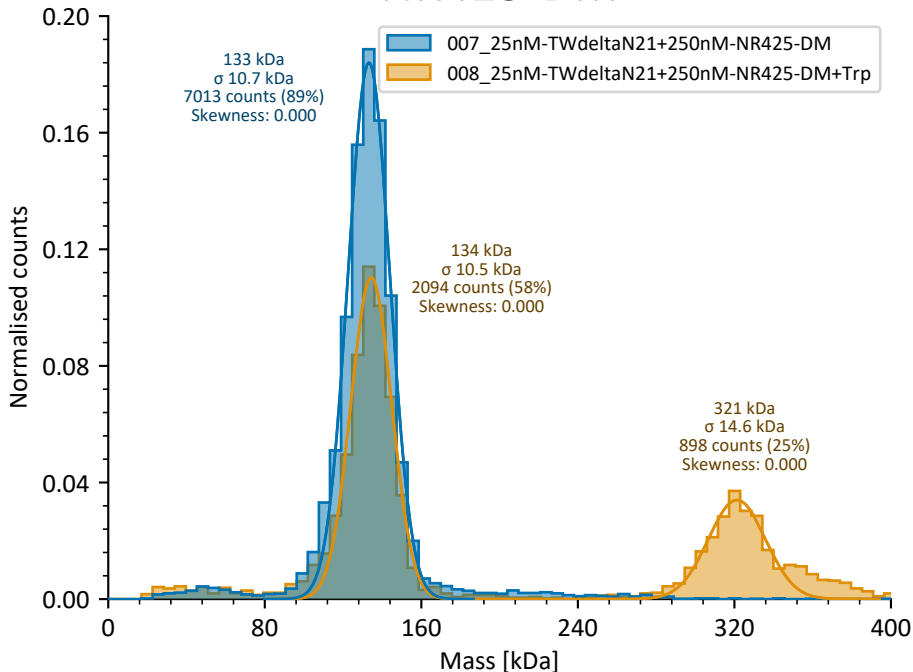

# NR428+Trp

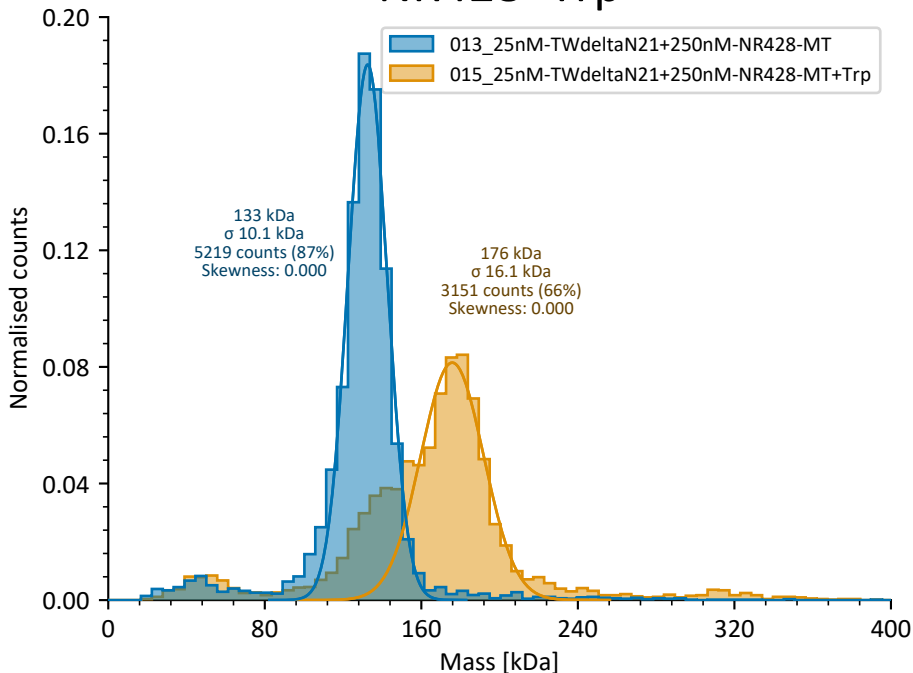

Supplement: Supplementary file 12 — Mass photometry output data. [file 41565_2026_2211_MOESM12_ESM.zip › Extended_Data_Figure_3_Source_Data/ExtendedFig3C_SourceData.pdf]

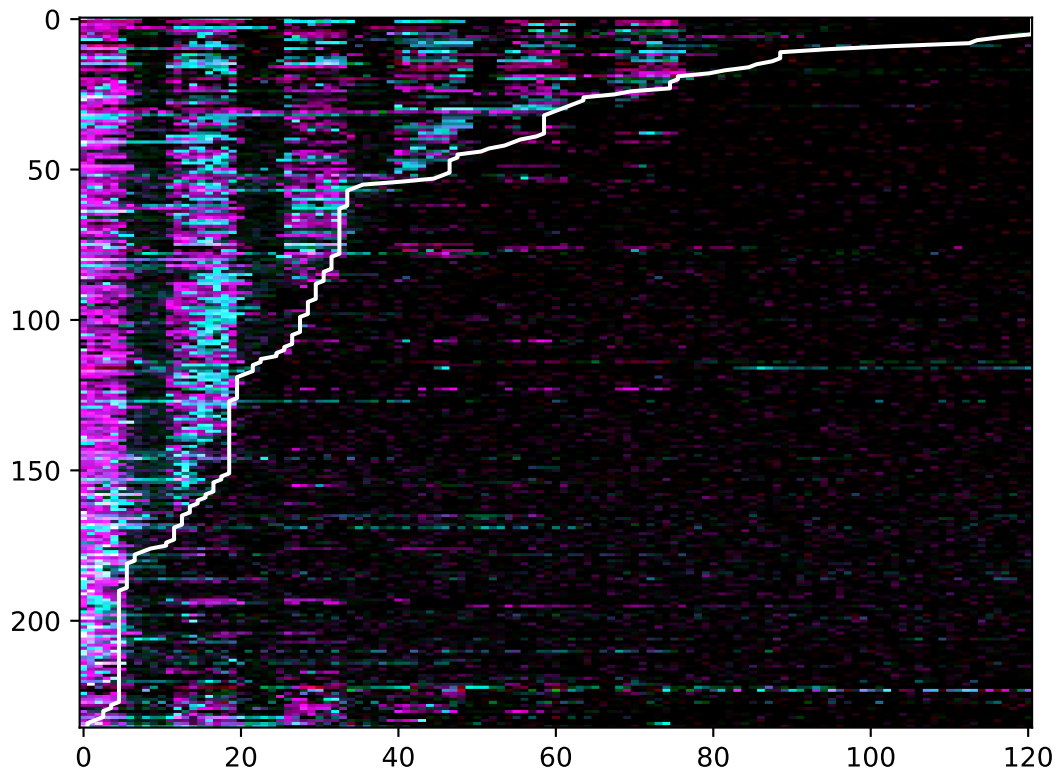

Supplement: Supplementary file 14 — Text files of single-molecule FRET and simulation output. [file 41565_2026_2211_MOESM14_ESM.zip › Extended_Data_Fig7_SourceData/Sim from exp 2.pdf]

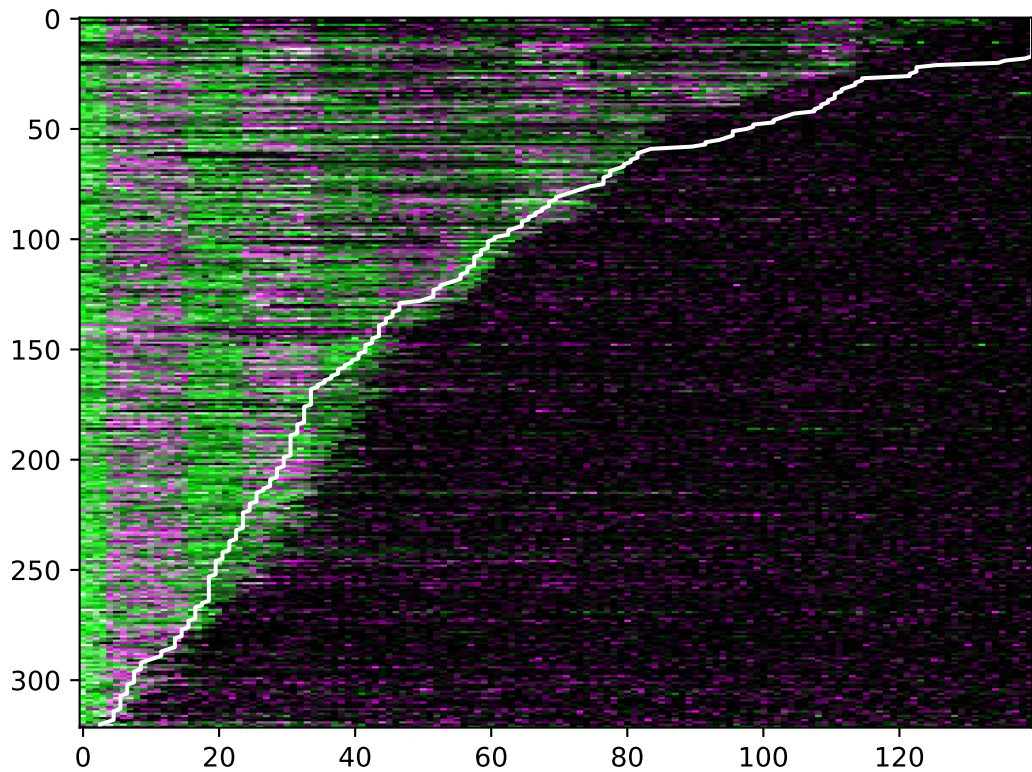

Supplement: Supplementary file 14 — Text files of single-molecule FRET and simulation output. [file 41565_2026_2211_MOESM14_ESM.zip › Extended_Data_Fig7_SourceData/Sim from exp 1.pdf]
